# Immunoregulation of macrophages by dynamic ligand presentation via ligand–cation coordination

**DOI:** 10.1038/s41467-019-09733-6

**Published:** 2019-04-12

**Authors:** Heemin Kang, Boguang Yang, Kunyu Zhang, Qi Pan, Weihao Yuan, Gang Li, Liming Bian

**Affiliations:** 1 Department of Biomedical Engineering, The Chinese University of Hong Kong, Hong Kong, 999077 China; 2 Department of Materials Science and Engineering, Korea University, Seoul, 02841 Republic of Korea; 3 Department of Orthopaedics & Traumatology, Faculty of Medicine, The Chinese University of Hong Kong, Prince of Wales Hospital, Shatin, Hong Kong 999077 China; 4 Stem Cells and Regenerative Medicine Laboratory, Lui Che Woo Institute of Innovative Medicine, Li Ka Shing Institute of Health Sciences, The Chinese University of Hong Kong, Prince of Wales Hospital, Shatin, Hong Kong 999077 China; 5 The CUHK-ACC Space Medicine Centre on Health Maintenance of Musculoskeletal System, Shenzhen Research Institute, The Chinese University of Hong Kong, Shenzhen, 518172 China; 6 Shenzhen Research Institute, The Chinese University of Hong Kong, Shenzhen, 518172 China; 7 China Orthopaedic Regenerative Medicine Group (CORMed), Hangzhou, Zhejiang 310058 China; 8 Centre for Novel Biomaterials, The Chinese University of Hong Kong, Hong Kong, 999077 China

## Abstract

Macrophages regulate host responses to implants through their dynamic adhesion, release, and activation. Herein, we employ bisphosphonate (BP)-coated gold nanoparticle template (BNP) to direct the swift and convertible formation of Mg^2+^-functional Mg^2+^-BP nanoparticle (NP) on the BP-AuNP surface via reversible Mg^2+^-BP coordination, thus producing (Mg^2+^-BP)-Au dimer (MgBNP). Ethylenediaminetetraacetic acid-based Mg^2+^ chelation facilitates the dissolution of Mg^2+^-BP NP, thus enabling the reversion of the MgBNP to the BNP. This convertible nanoassembly incorporating cell-adhesive Mg^2+^ moieties directs reversible attachment and detachment of macrophages by BP and EDTA, without physical scraping or trypsin that could damage cells. The swift formation of RGD ligand- and Mg^2+^-bifunctional RGD-Mg^2+^-BP NP that yields (RGD-Mg^2+^-BP)-Au dimer (RGDBNP) further stimulates the adhesion and pro-regenerative M2-type polarization of macrophages, both in vitro and in vivo, including rho-associated protein kinase. This swift and non-toxic dimer formation can include diverse bio-functional moieties to regulate host responses to implants.

## Abstract (web summary)

Control of macrophage adhesion and phenotype is important to biomaterial applications. Here, the authors report on the use of bisphosphonate coated gold nanoparticles by magnesium coordination for the controlled adhesion and polarisation of macrophages in vitro and in vivo and controlled cell release.

## Introduction

Macrophages are crucial immune cells that regulate immune systems, disease progression, and wound healing^1^. When macrophages are activated, they can be polarized into different phenotypes that elicit distinct immune functions, which are simply classified as pro-inflammatory M1-type or pro-regenerative M2-type. Macrophages also display a spectrum of activation states involving central transcriptional regulators that govern macrophage activation, and stimulus-specific regulators^2^. Macrophages play a key role in regulating foreign body response^3,4^. Foreign body response to implants is induced primarily by macrophages and foreign body giant cells that regulate the inflammatory and wound healing responses^4^. Macrophages that polarize into M2 phenotypes typically undergo fusion to form foreign body giant cells^4^. Macrophages and foreign body giant cells depending on their phenotypes over the time course secrete various cytokines and growth factors that coordinate foreign body response^4^. Hence, designing biomaterials with their cell-adhesive surface properties that are diversely convertible, can offer a powerful control over the foreign body responses to the implanted biomaterials by modulating the attachment and activation of macrophages^5^.

Macrophages actively associate with the extracellular matrix that modulates their attachment, detachment, and activation. Developing biomaterials with cell-adhesive functional moieties can enable the manipulation of reversible attachment and detachment of macrophages as well as their functional activation. The inclusion of Mg^2+^ moieties in biomaterials can effectively stimulate integrin binding and cellular attachment^6–8^. The RGD motif can act as an integrin-binding ligand to mediate the attachment of macrophages^9,10^. The detachment of macrophages has typically been achieved by physically scraping them from culture substrate surfaces^11–13^. However, this method can mechanically damage the macrophages, yielding detached macrophages in low viability^13^. Trypsin-ethylenediaminetetraacetic acid (EDTA) buffer has been commonly employed to detach cells from various substrate surfaces. However, trypsin is a proteolytic enzyme that cleaves cell surface receptors that carry out various cellular functions including cell attachment and spreading^14^. Thus, developing new biomaterials that can enable the non-toxic, efficient, and reversible attachment and detachment of macrophages without the use of physical scraping or proteolytic enzymes can offer unique advantages over those conventional methods. In addition, adhesive features in macrophages were reported to modulate their polarization phenotypes^15–18^. The spatial patterning of pre-assembled nanomaterials presenting various bio-functional motifs were reported to modulate the attachment or activation of immune cells^19–22^. Thus, it is desirable to develop materials that can manipulate the attachment and detachment of macrophages as well as their phenotypic polarization by dynamically presenting bio-functional moieties.

Developing biomaterials with bio-functional moieties that can be converted by an extrinsic trigger in vivo can manipulate the function of host macrophages to modulate host responses to the implants. Cell–material interactions have been manipulated by various physical and chemical trigger. For example, a magnetic field was used in our own recent studies to manipulate the tether mobility or continuous motion of RGD-coated superparamagnetic nanoparticles to control cell attachment^23,24^. Light was used to mediate the conversion between cell-adhesive and non-adhesive biomaterials via the release of RGD-conjugated DNA^25^ or photo-induced isomerization^26^ in vitro or to trigger the attachment of host macrophages in vivo by the photocleavage of RGD-caging molecule^10^. Enzymes were used as chemical trigger to modulate cell attachment by cleaving bio-functional moieties in vitro^27^. However, to the best of our knowledge, there have been no prior studies demonstrating the regulation of reversible attachment and detachment of macrophages, and their phenotypic polarization by an extrinsic trigger.

**Fig. 1:**
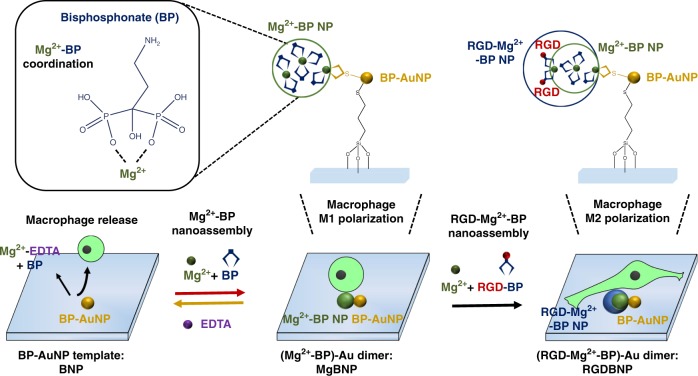
Summary of experimental procedures used in this study. Swift and convertible nano-formation was simply manipulated by the dynamic and reversible coordination of Mg^2+^ and bisphosphonate (BP), to regulate the attachment, detachment, and phenotypic polarization of macrophages. Mg^2+^ and BP assembled into cell-adhesive Mg^2+^-functional Mg^2+^-BP nanoparticle (NP), yielding (Mg^2+^-BP)-Au dimer (MgBNP, shown by a red arrow), whereas EDTA disassembled the Mg^2+^-BP NP, into non-adhesive “BNP” template (shown by a yellow arrow). RGD-BP and Mg^2+^ assembled into RGD- and Mg^2+^-bifunctional RGD-Mg^2+^-BP NP, resulting in (RGD-Mg^2+^-BP)-Au dimer (RGDBNP, shown by a black arrow), to further promote the attachment and M2-type polarization of macrophages

In this study, we utilize the swift and convertible dimer formation driven by the reversible Mg^2+^-bisphosphonate (BP) coordination by employing small molecules utilized in the clinics as the trigger, to manipulate the attachment, detachment, and polarization of macrophages (Fig. 1). We conjugate the BP-coated gold nanoparticle monomer template (BP-AuNP) (BNP) to a substrate. Supply of Mg^2+^ and BP leads to the formation of cell-adhesive Mg^2+^-BP nanoparticle (Mg^2+^-BP NP) on the BP-AuNP template, resulting in the growth of Mg^2+^-functional (Mg^2+^-BP)-Au dimer (MgBNP). Chelation of Mg^2+^ by EDTA disassembles the Mg^2+^-BP NP in the dimer, resulting in the reversion of the MgBNP to the BNP. This convertible process is performed with macrophages to enable their reversible attachment and detachment. We employ BP and EDTA, non-toxic small molecules, and thus supply RGD-conjugated BP, BP, and Mg^2+^ to direct the formation of RGD-Mg^2+^-BP NP, thus generating RGD- and Mg^2+^-bifunctional (RGD-Mg^2+^-BP)-Au dimer (RGDBNP), which promotes the attachment and M2-type polarization of macrophages. Biomaterials that can reversibly convert their cell-adhesive and non-adhesive property can culture macrophages in a reversible and non-toxic way or function as implants that direct the attachment and activation of host macrophages to modulate inflammatory or tissue-healing processes.

## Results

### Ligand–cation coordination drives cationic nanoassembly

The swift and convertible dimer formation was manipulated by small molecule-derived chemical trigger. The AuNP monomer was employed as a template. Dynamic light scattering analysis revealed that the size of the AuNPs was roughly 14 nm (Supplementary Fig. 1). We first conjugated AuNPs to thiolated glass substrate via gold-sulfur bonding and subsequently coated the AuNPs with thiolated BP via gold-sulfur bonding (Supplementary Fig. 2). We PEGylated and thus inactivated the remaining space of the substrate, not decorated with the BP-AuNPs. TEM image revealed a single layer of the BP-AuNPs (BNP) that uniformly decorated the substrate (Supplementary Fig. 3a). The decoration of the substrate with the AuNPs was confirmed by energy dispersive spectrum (EDS) of the BNP substrate (Supplementary Fig. 3b). We utilized BP-coated AuNP (BNP) on the substrate as the template to direct the formation of Mg^2+^-BP-NP on its surface, driven by the coordination between the Mg^2+^ ion and BP molecule (Supplementary Fig. 4). BP includes two phosphonate groups that strongly bind to divalent cations, including the Mg^2+^ ion, which can build a network with the help of hydrogen bonds^28^. Thus, we hypothesized that the BP ligand on the surface of the AuNP would direct the binding of Mg^2+^, which facilitates the localized formation of the Mg^2+^-BP NP driven by the Mg^2+^-BP coordination on the surface of the BP-AuNP.

**Fig. 2:**
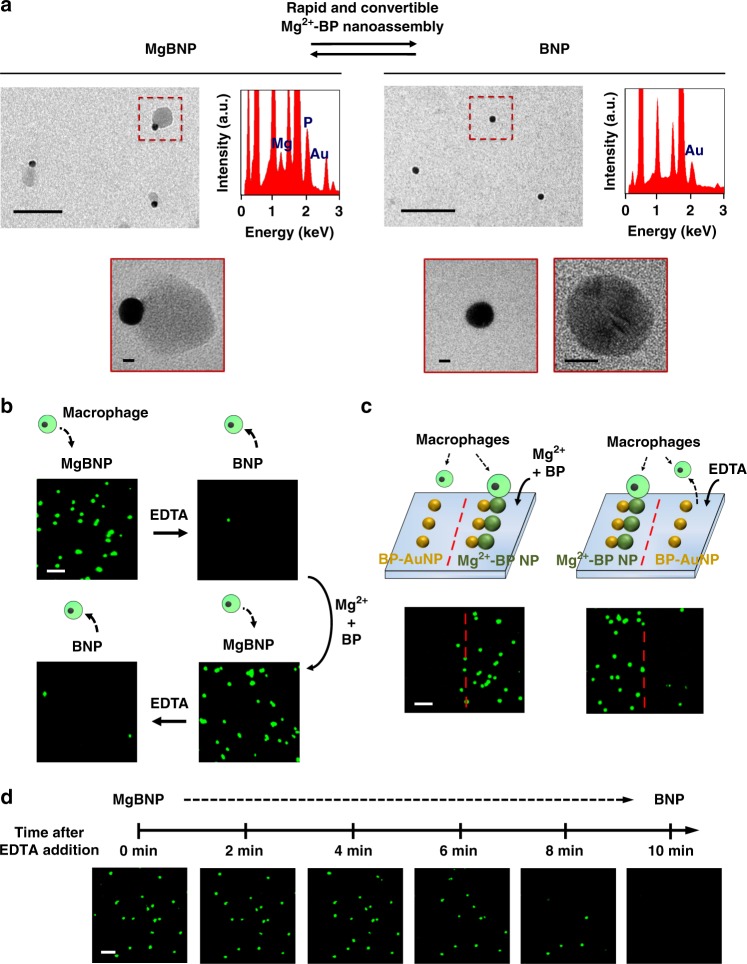
Convertible nano-formation manipulates attachment and detachment of macrophages. **a** Transmission electron microscopic (TEM) image and energy dispersive spectrum of a single layer of the MgBNP dimer or BNP template with reversibly assembled or disassembled Mg^2+^-BP NPs, respectively. Scale bars are 100 nm. For high-resolution TEM images, scale bars are 5 nm. Elemental Au represents the AuNPs, whereas elemental Mg and P indicate the newly formed Mg^2+^-BP NPs. **b** Fluorescently stained images of macrophages showing their repeated attachment and detachment, manipulated by the reversible formation and dissolution of the Mg^2+^-BP NPs. After the nano-formation by the coordination of Mg^2+^ and BP swiftly in 10 min, macrophages attached to the dimeric substrate for 12 h. Macrophages were detached during the dissolution induced by EDTA swiftly in 10 min. **c** Spatiotemporally directed patterning of macrophage attachment and detachment by the treatment of part of the substrate with Mg^2+^ and BP, or EDTA, respectively. **d** Time-dependent snapshots of fluorescently stained images showing macrophage detachment in real-time swiftly in 10 min by the nano-dissolution. Scale bars are 100 µm

We next employed the non-functional BP-AuNP-conjugated BNP substrate as a template for enabling the swift and convertible nano-formation under non-toxic conditions. The BNP substrate received Mg^2+^ (1 mM) and BP (1 mM) for enabling the growth of the Mg^2+^-BP NP on the BP-AuNP surface via Mg^2+^-BP coordination swiftly in 10 min, thus generating the (Mg^2+^-BP)-Au dimer (MgBNP). High-resolution TEM (HRTEM) image revealed a single layer of newly formed Mg^2+^-BP NP on the BP-AuNP surface in a dimeric MgBNP nanostructure (Fig. 2a). The formation of the Mg^2+^-BP NPs was confirmed by the EDS analysis with co-detection of the Mg and P element. The density of BP-AuNPs (BNP) was characterized to be roughly 79 nanoparticles/μm^2^ (Supplementary Fig. 3a). We determined that Mg^2+^-BP NPs formed on 81% of the BP-AuNP surface, resulting in a density of roughly 64 dimeric nanoparticles/μm^2^. We also treated the substrate with increased concentrations of Mg^2+^ (3 mM) and BP (3 mM) and found that the coverage of the BP-AuNP surface by the Mg^2+^-BP NPs slightly increased to 84%; however, this nano-formation produced the uncontrollable and heterogeneous size and shape of the Mg^2+^-BP NPs (Supplementary Fig. 5). We used the nano-formation condition with Mg^2+^ (1 mM) and BP (1 mM) in this study because it facilitated the homogenous, and controllable formation of the Mg^2+^-BP NPs. The nanoscale arrangements of bio-functional moieties, including their nanoparticle density and interparticle spacing^29,30^, influence cell attachment. Thus, in our study, we controlled the density of the Mg^2+^-functional Mg^2+^-BP NPs formed in dimers to direct efficient manipulation of the attachment and phenotypic polarization of macrophages. The MgBNP dimer was found to be stable under culture conditions for 7 days (Supplementary Fig. 6). The MgBNP dimeric substrate also received EDTA (1 mM) for dissolution of the Mg^2+^-BP NPs swiftly in 10 min by EDTA-based Mg^2+^ chelation, thereby directing their reversion to the BNP substrate (Supplementary Fig. 4). The BP-AuNPs were confirmed to be stable after they undergo the convertible formation and dissolution of the Mg^2+^-BP NPs (Fig. 2a).

### Cationic nanoassembly regulates macrophage attachment

The Mg^2+^ bio-functional moieties included in biomaterials stimulate integrin binding and cell attachment^6–8^. Hence, we hypothesized that convertibly manipulating the availability of cell-adhesive Mg^2+^ moieties in the Mg^2+^-BP NPs would convert cell-adhesive and non-adhesive property of biomaterials, thereby enabling the reversible attachment and detachment of macrophages. We next explored whether this convertible formation of Mg^2+^-BP NPs can direct the repeated attachment and detachment of macrophages. The (Mg^2+^-BP)-Au dimer (MgBNP) obtained by the formation of the Mg^2+^-BP NPs supported the attachment of macrophages, whereas the BNP to which they reverted after the swift dissolution of the Mg^2+^-BP NPs, effectively detached by roughly 89% of the attached macrophages, and this attachment and detachment were repeatable in two series (Fig. 2b and Supplementary Fig. 6). This indicates that the BP-AuNPs BNP template supported convertible formation and dissolution of the Mg^2+^-BP NPs that can specifically interact with macrophages, thereby enabling the reversible attachment and detachment of macrophages. As a control, we tested the formation of Ca^2+^-BP NPs or Sr^2+^-BP NPs on the surface of BP-coated AuNPs (BNP). The newly formed Ca^2+^-BP NPs or Sr^2+^-BP NPs revealed similar size to that of the Mg^2+^-BP NPs (Supplementary Fig. 8a). We then explored the adhesion of macrophages to the (Ca^2+^-BP)-Au or (Sr^2+^-BP)-Au dimers. The extent of macrophage adhesion to the (Ca^2+^-BP)-Au dimers was comparable with that of the (Mg^2+^-BP)-Au dimers and it was significantly higher than that to the (Sr^2+^-BP)-Au dimers (Supplementary Fig. 8b, c). This suggests that the possible activation of integrin-mediated adhesion of macrophages was metal ion-dependent^31^. In addition, lower MgBNP dimer density (20 dimers/μm^2^) was also prepared and the attachment of macrophages to this substrate was significantly less efficient than that to the substrate with higher MgBNP dimer density (64 dimers/μm^2^), which was thus employed in our study (Supplementary Fig. 9a, b). Spatial patterning of macrophage attachment was achieved by treating the part of the BNP substrate with Mg^2+^ (1 mM) and BP (1 mM) swiftly in 10 min (Fig. 2c). Conversely, spatially regulated detachment of the attached macrophages was achieved by treating the part of the MgBNP dimeric substrate with EDTA (1 mM) swiftly in 10 min. The detachment of the attached macrophages swiftly in 10 min by the EDTA-directed dissolution of the Mg^2+^-BP NPs, was monitored with confocal microscopy (Fig. 2d, Supplementary Fig. 10, and Supplementary Movie 1). Taken together, the convertible formation and dissolution of the Mg^2+^-BP NPs spatially and effectively directed the repeated attachment and detachment of macrophages.

We next explored the viability of the detached macrophages. Roughly 93% of the detached macrophages were viable, and the detachment of viable macrophages was repeatable in two series, confirming highly non-toxic property of this small molecule-derived trigger that enabled the detachment of macrophages (Supplementary Fig. 11a, b). BP is not freely permeable to cell membrane due to negatively charged phosphonate group, but can undergo endocytosis^32^. A prior literature showed that BP can exert cytotoxicity to macrophages after macrophages are exposed to it for a prolonged period, typically 48 h^32^. Thus, we set a limit to the exposure of macrophages to BP within 10 min to minimize its cytotoxicity. As a result, both the attached and detached macrophages were highly viable after their limited exposure to BP. As control experiments, we tested the role of EDTA in mediating macrophage detachment from the MgBNP dimeric substrate. The EDTA-specific control over the macrophage detachment was confirmed by minimal detachment of the macrophages attached to the MgBNP dimeric substrate by EDTA-deficient buffer (Supplementary Fig. 12a, b). The AuNP-decorated substrate was fabricated by directly conjugating the thiolated RGD peptide to the AuNPs, which were conjugated to the substrate (Supplementary Fig. 13a). This RGD-AuNP-conjugated substrate, which supported the attachment of macrophages, was treated with trypsin-EDTA, EDTA, or physical scraping (Supplementary Fig. 13b). Without the EDTA-directed dissolution of the Mg^2+^-BP NPs, the detachment of macrophages by trypsin-EDTA or EDTA was not efficient, with only roughly 11–13% of the detached cells (Supplementary Fig. 13b, c). This indicates that the efficient detachment of macrophages from the MgBNP dimeric substrate is predominantly attributed to the EDTA-directed dissolution of the Mg^2+^-BP NPs (Fig. 2b and Supplementary Fig. 7). In addition, the detachment of macrophages by physical scraping was highly efficient but resulted in the low viability (62%) of the detached macrophages (Supplementary Fig. 13b–d). Taken together, these findings indicate that the non-toxic, efficient, and reversible attachment and detachment of macrophages induced by this convertible formation of the Mg^2+^-BP NPs without the use of physical scraping or proteolytic enzyme, offer unique advantages for applications in culturing and studying macrophages.

### Cationic nanoassembly supports dynamic ligand presentation

**Fig. 3:**
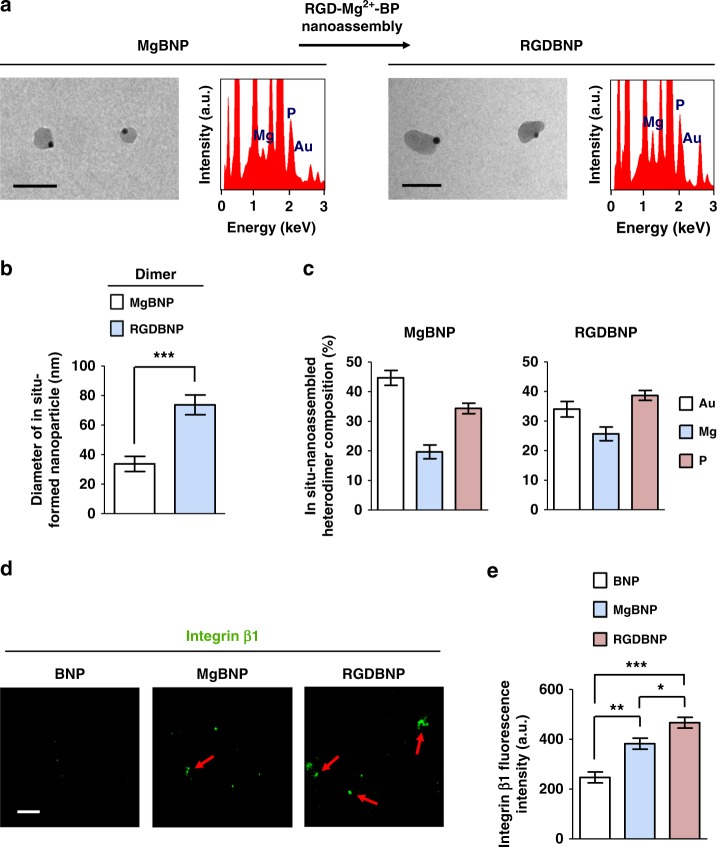
Swift dimer formation promotes the binding of integrin β1. **a** Transmission electron microscopy image and energy dispersive spectra of a single layer of the MgBNP dimer or RGDBNP dimer. Scale bars are 100 nm. **b** Approximate size and **c** content of Mg^2+^-BP NPs in the MgBNP dimer or in the RGDBNP dimer. Data are means ± s.d. (*n* = 10). ***P < 0.001 (two-tailed Student’s *t*-test). The content of the Mg^2+^-BP NPs is shown to compare relative amounts of elemental Mg and P in the Mg^2+^-BP NPs to that of elemental Au in the AuNPs, in the dimer. Data are means ± s.e.m. (*n* = 3). **d** Fluorescent images immunostained for integrin β1 bound to the substrate decorated with BNP, MgBNP dimer, or RGDBNP dimer, and **e** the determined fluorescence signal intensities. Red arrows represent substrate-bound integrin clusters. Scale bar is 100 µm. Data are means ± s.e.m. (*n* = 3). *P < 0.05, **P < 0.01, ***P < 0.001 (ANOVA)

We next explored whether we can enable the complex formation of nanoparticles including both the cell-adhesive ligand, RGD peptide and Mg^2+^ bio-functional moieties to further stimulate integrin binding with integrin-binding RGD ligand, and subsequent macrophage attachment. To this end, we synthesized RGD-conjugated BP (RGD-BP) and treated the MgBNP substrate with RGD-BP, Mg^2+^, and BP swiftly in 10 min to induce the swift formation of RGD- and Mg^2+^-bifunctional RGD-Mg^2+^-BP NPs that generates (RGD-Mg^2+^-BP)-Au dimer (RGDBNP) (Supplementary Fig. 14). TEM image revealed that a single layer of the RGDBNP dimer following the formation of the RGD-Mg^2+^-BP NPs on the surface of most of the MgBNP dimers (Fig. 3a). This formation of the RGD-Mg^2+^-BP NPs contributed to considerable elevation in the diameter of the Mg^2+^-BP NPs from 33.7 nm in the MgBNP dimers to 73.7 nm in the RGDBNP dimers (Fig. 3b). EDS analysis revealed considerable elevation in the relative compositions of elemental Mg and P to elemental Au, confirming the increase in the content of the Mg^2+^-BP NPs in the RGDBNP dimer (Fig. 3a, c). We next explored the binding of integrin β1 to the dimer formation. The extent of integrin β1 binding was significantly greater in the MgBNP and RGDBNP dimers than in the “BNP” (Fig. 3d, e). In addition, RGDBNP dimer showed significantly higher binding of integrin β1 than the MgBNP dimer.

**Fig. 4:**
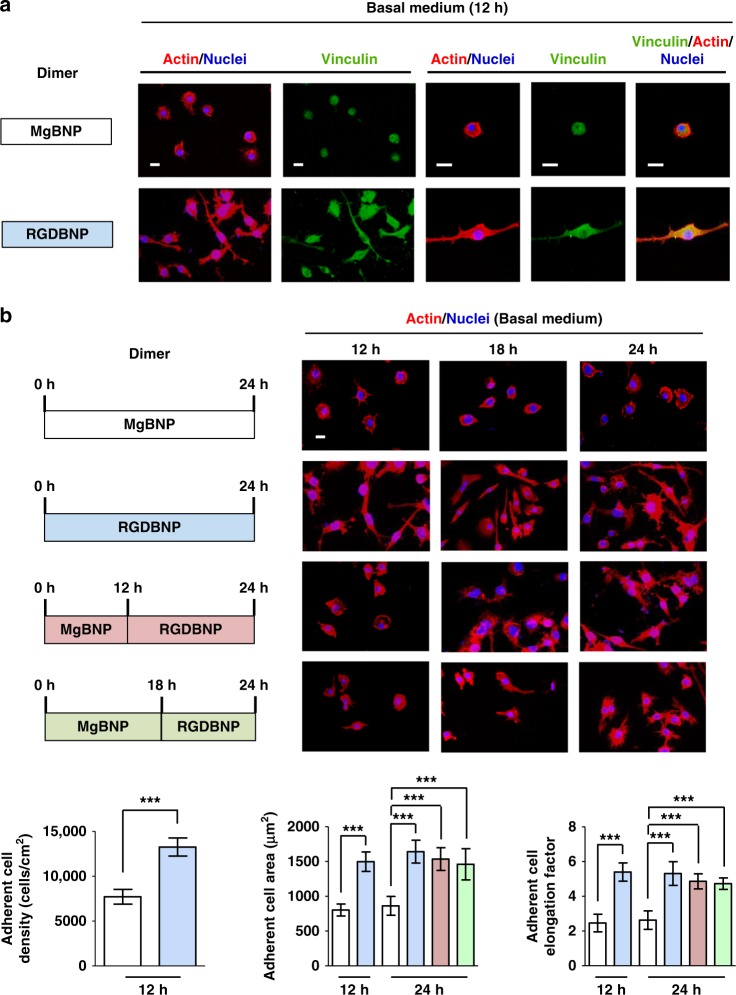
The dimer formation directs the time-controlled adhesion of macrophages. **a** Fluorescent images of macrophages immunostained for vinculin (in green), actin (in red), and nuclei (in blue) after 12 h under culture. **b** Fluorescent images of macrophages stained for actin (in red) and nuclei (in blue) over the course of culture time (12, 18, or 24 h) and the determined representative densities, areas, and elongation factors of the attached macrophages. The dimer formation with the Mg^2+^-BP NPs (MgBNP dimer) or the RGD-Mg^2+^-BP NPs (RGDBNP dimer) was induced after 0 h under culture. The time-manipulated dimer formation was induced with Mg^2+^-BP NP formation after 0 h, followed by RGD-Mg^2+^-BP NP formation after 12 h or 18 h (MgBNP-RGDBNP 12 h or MgBNP-RGDBNP 18 h dimer). Scale bars are 50 µm. Data are means ± s.d. (*n* = 4 for densities and *n* = 10 for areas and elongation factors). ***P < 0.001 (two-tailed Student’s *t*-test or ANOVA)

We next explored whether this enhanced integrin β1 binding to the RGD-presenting dimer formation can stimulate the time-controlled formation of the adhesive features in macrophages. We explored the attachment of macrophages to the substrate after inducing the dimer formation of the Mg^2+^-BP NPs (MgBNP dimer) or RGD-Mg^2+^-BP NPs (RGDBNP dimer) after 0 h under culture. After 12 h under culture with basal medium, significantly higher density of attached macrophages (72% more adherent cells) was observed in the RGD- and Mg^2+^-bifunctional RGDBNP dimer than in the Mg^2+^-functional MgBNP dimer (Fig. 4a, b). We carried out control experiments by inducing the subsequent formation of Mg^2+^-BP NPs on the MgBNP dimeric substrate with or without RGD-BP. As a result, only the subsequent formation of the Mg^2+^-BP NPs with RGD-BP significantly enhanced macrophage attachment, when compared with that without the subsequent formation, whereas the subsequent formation without RGD-BP did not significantly enhance macrophage attachment (Supplementary Fig. 15a, b). We tested the functionality of RGD peptide by utilizing RGE peptide as a mutated control peptide^33^. We found that the subsequent formation of the Mg^2+^-BP NPs with RGE-BP did not significantly promote macrophage adhesion, when compared with the MgBNP dimer, thereby confirming the functionality of the RGD peptide to promote macrophage adhesion (Supplementary Fig. 16a, b). We also found that the subsequent formation of the Mg^2+^-BP NPs with cyclic RGD-BP or linear RGD-BP both significantly promoted macrophage adhesion, when compared with the MgBNP dimer, indicating that both cyclic and linear RGD peptides can promote macrophage adhesion (Supplementary Fig. 16a, b). These findings indicate the efficacy and functionality of the RGD-BP in the dimer to enhance integrin binding, thereby facilitating macrophage adhesion. In addition, we analyzed the adhesive features of macrophages by measuring their areas and elongation factors, which may modulate their phenotypic polarization. The attached macrophages to the RGDBNP dimer revealed predominant actin formation and vinculin expression, confirming that the robust attachment of macrophages was facilitated by the inclusion of the RGD-BP (Fig. 4a). In addition, significantly greater area (by 87%) and higher elongation factor (by 120%) were observed in the RGDBNP dimer than in the MgBNP dimer (Fig. 4b). Macrophages exhibit their time-resolved dynamics in their attachment and polarization that elicit distinct functions^34^. Thus, we next explored the time-controlled tuning of dimer formation by inducing the Mg^2+^-BP NP formation first (at 0 h) and then the RGD-Mg^2+^-BP NP formation after 12 h or 18 h (MgBNP-RGDBNP 12 h or MgBNP-RGDBNP 18 h dimer), to explore the possibility of time-manipulated attachment of macrophages. Strikingly, the delayed formation of the RGD-Mg^2+^-BP NPs after 12 or 18 h under cell culture condition (MgBNP-RGDBNP 12 h and MgBNP-RGDBNP 18 h dimers) significantly increased the area and elongation factor of attached macrophages after 24 h, when compared with those of the MgBNP dimer (Fig. 4b). These findings confirm the utility of the dimer formation for the time-resolved manipulation of macrophage adhesion.

### Dynamic ligand presentation controls macrophage polarization

The adhesive features in macrophages were reported to regulate the phenotypic polarization of macrophages together with soluble inducers, such as M1-polarizing or M2-polarizing inducers^15,18^. Therefore, we next explored whether the formation of the RGD-presenting dimer that stimulated the development of adhesive features in macrophages, can further enhance the M2-type polarization of macrophages. The time-controlled phenotypic polarization of attached macrophages was explored after 36 h under culture (the first 12 h under culture with basal medium when macrophages attach, followed by 24 h under culture with M1-type-polarizing or M2-type-polarizing medium). The detached macrophages obtained via the dissolution of Mg^2+^-BP NPs re-adhered and maintained their polarization capacity as evidenced by their efficient polarization into M1-type or M2-type under the culture with M1-type- or M2-type-polarizing medium, respectively (Supplementary Fig. 17). We induced the dimer formation of the Mg^2+^-BP NPs or the RGD-Mg^2+^-BP NPs after 0 h under culture. We also induced the Mg^2+^-BP NP formation after 0 h, followed by the RGD-Mg^2+^-BP NPs formation after 12 h, for time-manipulated dimer formation.

**Fig. 5:**
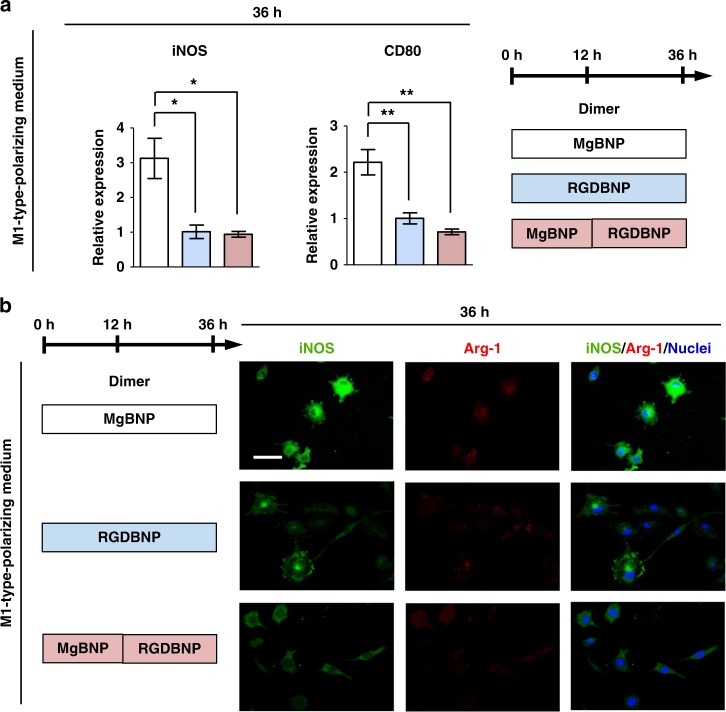
The dimer formation presenting RGD-BP inhibits M1-type polarization of macrophages. **a** Expression levels of M1-type markers (iNOS and CD80 genes) determined by RT-qPCR, and **b** fluorescent images of macrophages immunostained for iNOS (in green), Arg-1 (in red), and nuclei (in blue) at 36 h under culture (the first 12 h under culture with basal medium, followed by 24 h under culture with M1-type-polarizing medium). The dimer formation of the Mg^2+^-BP NPs (MgBNP dimer) or the RGD-Mg^2+^-BP NPs (RGDBNP dimer) was induced after 0 h under culture. The time-controlled tuning of dimer formation was induced with the Mg^2+^-BP NP formation after 0 h, followed by the RGD-Mg^2+^-BP NP formation after 12 h (MgBNP-RGDBNP dimer). Data are means ± s.e.m. (*n* = 3). *P < 0.05, **P < 0.01 (ANOVA). Scale bar is 50 µm

After macrophages were cultured under M1-type-polarizing medium, we carried out a western blotting analysis to detect M1-type marker (iNOS expression) or M2-type marker (Arg-1 expression). The iNOS expression was significantly suppressed in the RGD- and Mg^2+^-bifunctional RGDBNP dimer, when compared with that in the Mg^2+^-functional MgBNP dimer, and the Arg-1 expression was not considerably detected in both dimers (Supplementary Fig. 18a, b). We next analyzed the expression levels of M1-type markers (*iNOS* and *CD80* genes) by reverse transcription-quantitative polymerase chain reaction (RT-qPCR) after 36 h under culture. The levels of both *iNOS* expression (by 68% and 70%, respectively) and *CD80* expression (by 55% and 68%, respectively) were significantly downregulated in the RGDBNP and MgBNP-RGDBNP dimers, when compared with their expression in the MgBNP dimer (Fig. 5a). The inhibition of M1-type polarization by the RGD-presenting dimer was also corroborated with immunostaining for M1-type (iNOS protein) and M2-type (Arg-1 protein) marker. The MgBNP dimer yielded considerably higher iNOS fluorescence signal in most cells than the “RGDBNP” and MgBNP-RGDBNP dimers (Fig. 5b). We also found that the secretion of pro-inflammatory cytokines, such as TNF-α, IL-1β, and IL-6, was reduced in the RGDBNP dimer when compared with the MgBNP dimer, with no significant difference in the secretion of anti-inflammatory cytokines, such as IL-10^35^ (Supplementary Fig. 19). These findings confirm that the RGD-presenting dimer formation can temporally suppress the M1-type polarization of attached macrophages.

**Fig. 6:**
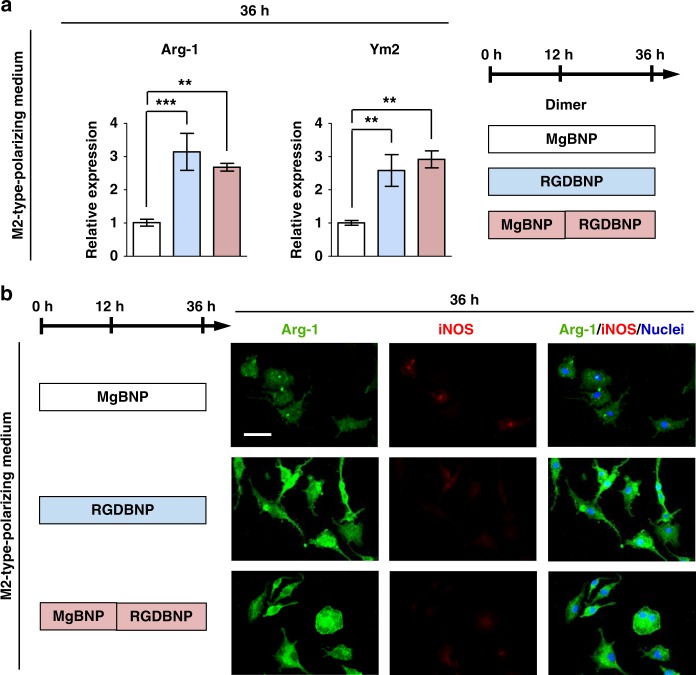
RGD-presenting dimer formation promotes M2-type polarization of macrophages. **a** Expression levels of M2-type markers (Arg-1 and Ym2 genes) determined by RT-qPCR, and **b** fluorescent images of macrophages immunostained for Arg-1 (in green), iNOS (in red), and nuclei (in blue) after 36 h under culture (the first 12 h under culture with basal medium, followed by 24 h under culture with M2-type-polarizing medium). The dimer formation with the Mg^2+^-BP NPs (MgBNP dimer) or the RGD-Mg^2+^-BP NPs (RGDBNP dimer) was directed after 0 h under culture. The time-controlled dimer formation was directed with the Mg^2+^-BP NP formation after 0 h, followed by the RGD-Mg^2+^-BP NP formation after 12 h (MgBNP-RGDBNP dimer). Data are means ± s.e.m. (*n* = 3). **P < 0.01, ***P < 0.001 (ANOVA). Scale bar is 50 µm

Conversely, after macrophages were cultured under M2-type-polarizing medium, a western blotting analysis revealed that the RGDBNP dimer showed significantly higher levels of Arg-1 expression than in the MgBNP dimer, whereas iNOS expression was not considerably detected in both dimers (Supplementary Fig. 20a, b). In addition, significantly upregulated levels of *Arg-1* expression (by 211% and 165%, respectively) and *Ym2* expression (by 157% and 190%, respectively) were observed in the RGDBNP and MgBNP-RGDBNP dimers, when compared with those in the MgBNP dimer (Fig. 6a). Immunostaining for Arg-1 showed considerably higher fluorescence signal in most cells in both the RGDBNP and MgBNP-RGDBNP dimers than in the MgBNP dimer (Fig. 6b). We further confirmed by flow cytometry that the expression of Arg-1 was significantly higher in the RGDBNP dimer than the MgBNP dimer, with no significant difference in the iNOS expression (Supplementary Fig. 21). We also found that the secretion of anti-inflammatory cytokines, such as IL-10, was enhanced in the RGDBNP dimer with no significant differences in the secretion of pro-inflammatory cytokines, such as IL-12, IL-1β, and IL-6 (Supplementary Fig. 22). In addition, we cultured macrophages for 7 days under a medium containing IL-4 and IL-13 cytokines, stimulators for macrophage fusion. We found that MgBNP dimer appeared to inhibit macrophage fusion as compared with that in the RGDBNP dimer (Supplementary Fig. 23a, b). These findings confirm that the pervasive formation of the adhesive features in macrophages stimulated by the RGD-presenting nano-formation, further promoted their M2-type polarization but suppressed their M1-type polarization.

**Fig. 7:**
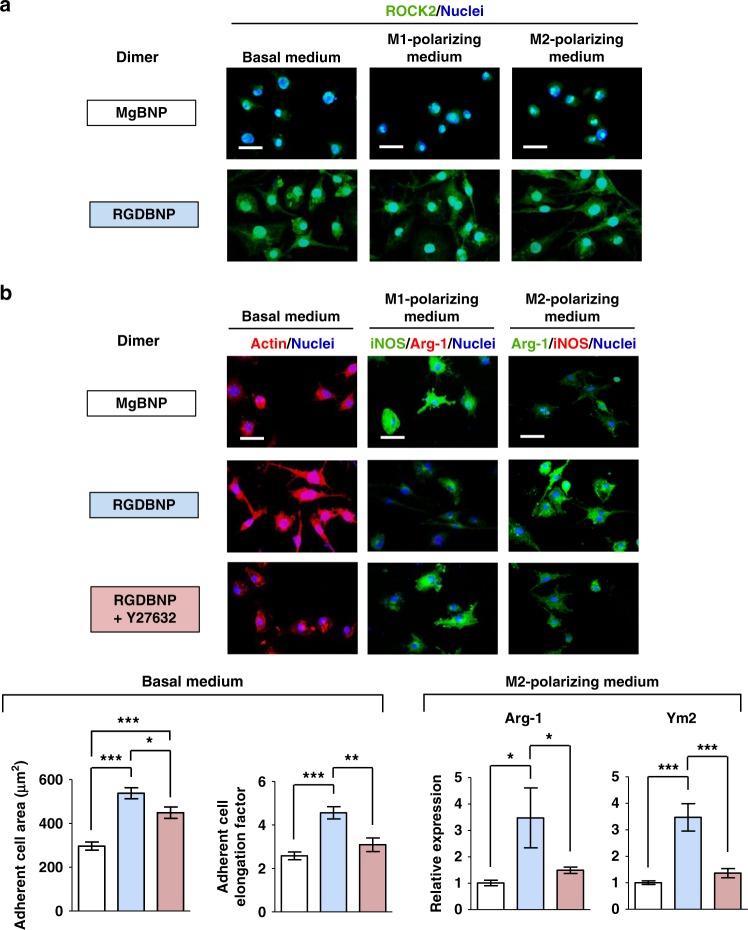
Nano-formation-controlled attachment and polarization of macrophages include ROCK. **a** Fluorescent images of macrophages immunostained for ROCK2 (in green) and nuclei (in blue) in macrophages after 24 h after culture with basal medium, 36 h after M1-polarizing culture, or 36 h after M2-polarzing culture. **b** Fluorescent images of macrophages stained for actin (in red) and nuclei (in blue) under basal medium culture for 24 h, iNOS (in green), Arg-1 (in red), and nuclei (in blue) under M1-polarizing culture for 36 h, or Arg-1 (in green), iNOS (in red), and nuclei (in blue) under M2-polarzing culture for 36 h. The dimer formation with the Mg^2+^-BP NPs (MgBNP dimer) or the RGD-Mg^2+^-BP NPs (RGDBNP dimer) was directed after 0 h under culture. Cells in the “RGDBNP” dimer was also cultured with the ROCK-inhibiting drug, Y27632 (RGDBNP + Y27632). Scale bars are 50 µm. The representative areas and elongation factors of attached macrophages were determined after culture under basal medium. Data are means ± s.e.m. (*n* = 10). Expression levels of M2-type markers (Arg-1 and Ym2 genes) determined by RT-qPCR, after cell culture under M2-type-polarizing medium. Data are means ± s.e.m. (*n* = 3). *P < 0.05, **P < 0.01, ***P < 0.001 (ANOVA)

We next explored how the adhesive features of macrophages may regulate their phenotypic polarization. Rho-associated protein kinase (ROCK) is a key mediator that regulates adhesive structures, such as the actin cytoskeletal organization and contractility. ROCK2 mediates the M2-type polarization of macrophages and functions as a molecular switch in macrophages between their M2-type and M1-type^36^. We cultured macrophages under basal medium or, M1- or M2-polarizing medium and found high fluorescence signal of ROCK2 in the RGDBNP dimer than in the MgBNP dimer (Fig. 7a). We next inhibited ROCK with the Y27632 pharmacological agent and explored the adhesive structures and phenotypic polarization of macrophages. Significantly higher areas and elongation factors of attached macrophages observed in the RGDBNP dimer were abrogated by the ROCK-inhibiting drug (Fig. 7b). Interestingly, the RGD-presenting dimer-stimulated M2-type polarization of attached macrophages observed in the RGDBNP dimer under M2-polarizing culture was significantly hindered by the ROCK-inhibiting drug (Fig. 7b), without significant difference in the M1-type polarization (Supplementary Fig. 24). In addition, the attached macrophages in the RGDBNP dimer showed the expression of M1-type polarization by the ROCK-inhibiting drug under M1-polarizing culture, which was not observed without the ROCK-inhibiting drug, without significant difference in the M2-type polarization in the three groups (Fig. 7b and Supplementary Fig. 25). These findings suggest that the enhanced attachment and M2-type polarization of macrophages achieved by the RGD-presenting nano-formation included ROCK, consistent with a prior literature^36^.

### Ligand nanoassembly regulates host macrophage polarization

**Fig. 8:**
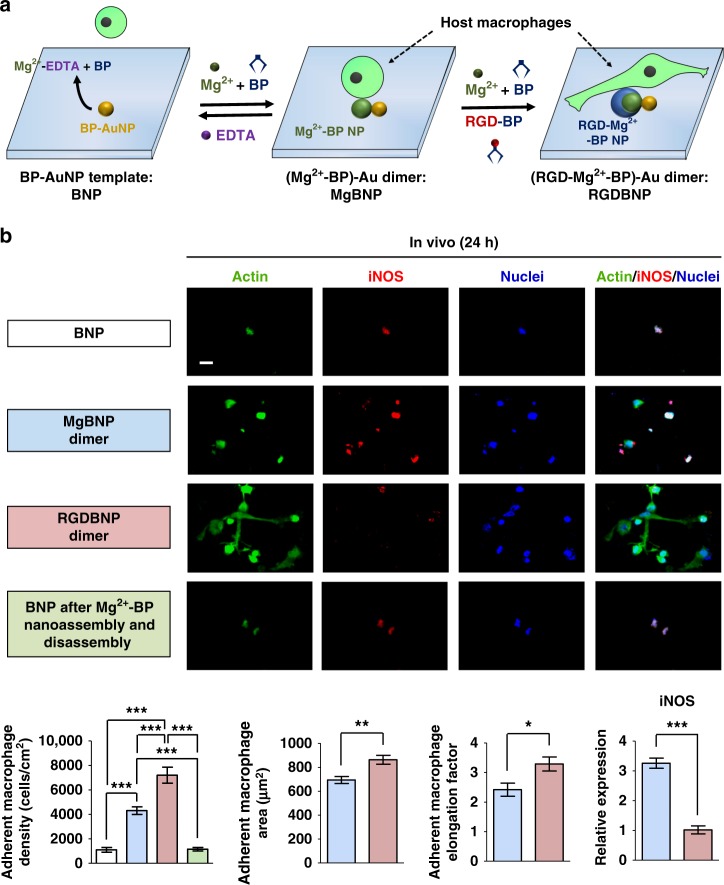
The convertible dimer formation manipulates M1-type macrophages in vivo. **a** Schematic representation of the BNP substrate implanted into a subcutaneous pocket, which was then subjected to reversible dimer formation by the administration of BP, Mg^2+^, EDTA, or RGD-BP. Five mice were used per group. **b** Fluorescent images of the attached host cells, immunostained for actin (in green), iNOS (in red), and nuclei (in blue) at 24 h post implantation. Scale bar is 20 µm. The representative densities, areas, and elongation factors of the attached host macrophages were determined, which were randomly chosen, at 24 h post implantation. Data are means ± s.e.m. (*n* = 4 for densities and *n* = 10 for areas and elongation factors). Expression levels of M1-type marker (iNOS gene) determined by RT-qPCR, for the attached host cells, which were randomly chosen, at 24 h post implantation. Data are means ± s.e.m. (*n* = 3). *P < 0.05, **P < 0.01, ***P < 0.001 (ANOVA or two-tailed Student’s *t*-test)

Implanted biomaterials trigger host responses, in which functional activation of macrophages plays a key role^4^. Hence, it is a vital requirement to modulate the attachment and functional states of macrophages to achieve desirable immune responses to the implants. To this end, we explored whether the convertible dimer nano-formation can convert their cell-adhesive and non-adhesive surface property in vivo to regulate the attachment and activation of host macrophages. We employed the non-functional PEGylated BNP substrate as the implant into a subcutaneous pocket and then administered small molecules used in the clinics, such as BP^37^ or EDTA^38^, or ions (Mg^2+^), for non-toxic and reversible conversion between cell-adhesive and non-adhesive implant surface (Fig. 8a). At 24 h post implantation, we investigated the recruitment, attachment, and phenotypic polarization of host macrophages on the surface of implants. We first analyzed cytoskeletal structure of host macrophages by actin and their phenotypic polarization into either M1-type or M2-type states.

**Fig. 9:**
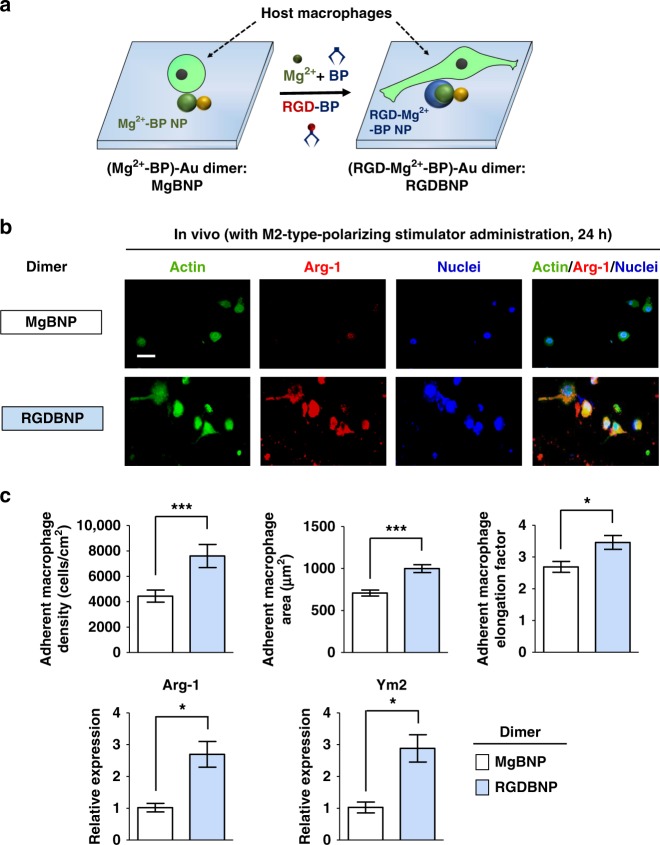
The dimer formation facilitates M2-type macrophages in vivo with M2 stimulators. **a** Schematic representation of the “BNP” substrate implanted into a subcutaneous pocket, which was then subjected to dimer formation by the administration of BP, Mg^2+^, or RGD-BP followed by M2-type-polarizing inducers (IL-4 and IL-13 cytokines). Five mice were used per group. **b** Fluorescent images of the attached host cells, immunostained for actin (green), Arg-1 (red), and nuclei (blue) at 24 h post implantation. Scale bar is 20 µm. **c** The representative densities, areas, and elongation factors of the attached macrophages were determined, which were randomly chosen, at 24 h post implantation. Data are means ± s.d. (*n* = 4 for densities). Data are means ± s.e.m. (*n* = 10 for areas and elongation factors). Expression levels of M2-type markers (Arg-1 and Ym2 genes) determined by RT-qPCR, for the attached host cells, which were randomly chosen, at 24 h post implantation. Data are means ± s.e.m. (*n* = 3). *P < 0.05, ***P < 0.001 (two-tailed Student’s *t*-test)

At 24 h post implantation, the attachment of the host macrophages was markedly higher in the MgBNP and RGDBNP dimers (by 293% and 557%, respectively) than the BNP (Fig. 8b). This indicates that the implant surface decorated with non-functional BNP and PEGylation suppressed host cell attachment to the non-adhesive surface. Interestingly, conversion from the BNP to the MgBNP dimer and then reverting to the BNP also efficiently minimized host cell attachment, confirming that the nano-formation and dissolution were converted, which yielded cell-adhesive and non-adhesive surfaces in vivo, respectively (Fig. 8b). In addition, the RGDBNP dimer exhibited higher density, area, and elongation factor of the attached macrophages as well as less intense iNOS fluorescence signal, but higher levels of Arg-1 signal than the MgBNP dimer (Fig. 8b and Supplementary Fig. 26). We also explored the phenotypic polarization of the host cells by RT-qPCR according to a prior literature^5^. The levels of both *iNOS* expression (by 220%) and *CD80* expression (by 174%) were upregulated in the MgBNP dimer, when compared with their expression in the RGDBNP dimer (Fig. 8b and Supplementary Fig. 27). The levels of *Arg-1* and *Ym2* expression did not significantly differ in both dimers (Supplementary Fig. 27). These collectively indicate the attachment of host macrophages was enhanced by the RGD-presenting dimer, but their M1-type polarization was suppressed. In addition to macrophages, neutrophils are involved in the innate immune responses to implants. The RGDBNP dimer showed higher attachment density of NIMP-R14-positive host neutrophils, when compared with the MgBNP dimer (Supplementary Fig. 28a, b). We next explored whether the dimer formation presenting RGD-BP can function in concert with M2-type-polarizing inducers (IL-4 and IL-13 cytokines) to further induce the pro-regenerative M2-type polarization of the attached host macrophages. We administered BP, Mg^2+^, or RGD-BP and then the M2-type-polarizing stimulators (Fig. 9a). With the M2-type-polarizing stimulators, the RGDBNP dimer showed higher adhesion of host macrophages as well as strongly positive Arg-1 fluorescence signal, but low iNOS signal (Fig. 9b, c and Supplementary Fig. 29). Contrastively, the MgBNP dimer showed a mixture of Arg-1-positive or iNOS-positive cells. The levels of both *Arg-1* expression (by 164%) and *Ym2* expression (by 181%) were upregulated in the RGDBNP, when compared with their expression in the MgBNP dimer (Fig. 9c). The levels of *iNOS* and *CD80* expression were not significantly different in both dimers (Supplementary Fig. 30). The RGDBNP dimer showed higher attachment density of host neutrophils, when compared with the MgBNP dimer (Supplementary Fig. 31). These collectively indicate that the attachment and M2-type polarization of host macrophages were both enhanced by the RGD-presenting dimer in concert with M2-type-polarizing stimulators.

Macrophages can fuse to form foreign body giant cells, which regulate foreign body response. To explore the fusion of macrophages after longer implantation period (10 days), we prepared and implanted AuNP-conjugated PEGDA-based hydrogels as the BNP substrate (Supplementary Fig. 32a). We formed the MgBNP or RGDBNP substrates in situ, and then administered M2-polarizing cytokines. We found that the RGDBNP substrate stimulated the fusion of host macrophages resembling foreign body giant cells as compared with the MgBNP substrate (Supplementary Fig. 32b, c). These findings indicate that the RGD-presenting dimer is capable of promoting the adhesion, M2 polarization, and fusion of host macrophages in vivo, which may regulate foreign body response to the implants. Our findings suggest that the non-toxic manipulation of convertible dimer formation can potentially regulate inflammation or pro-healing host responses.

## Discussion

In summary, we demonstrated the convertible dimer formation presenting integrin-binding cell-adhesive Mg^2+^ and RGD moieties via dynamic Mg^2+^-BP coordination. The nano-formation and dissolution, which can swiftly convert between Mg^2+^-functional (Mg^2+^-BP)-Au dimer (MgBNP) and Mg^2+^-deficient AuNP monomer (BNP), spatiotemporally manipulated reversible attachment and detachment of macrophages by utilizing small molecules used in the clinics, including BP and EDTA. We showed the reversible attachment and detachment of macrophages manipulated by the convertible nano-formation and dissolution, and this can be used to reversibly and cytocompatibly culture any cell types without the need for physical scraping or enzymes, such as trypsin, which could damage cells. The formation of RGD- and Mg^2+^-bifunctional (RGD-Mg^2+^-BP)-Au dimer (RGDBNP) further enhanced the attachment and M2-type polarization of macrophages, including ROCK signaling, whereas the “MgBNP” dimer-stimulated M1-type polarization of macrophages. This convertible dimer formation enables complex presentation of various bio-functional moieties that could potentially facilitate tissue engineering by regulating host responses to the implants.

## Methods

### Bisphosphonate-AuNP (BNP)-coated substrate

AuNPs were synthesized by boiling HAuCl_4_·3H_2_O (0.88 mM) in deionized (DI) water (20 mL) with vigorous stirring first. To this solution, 1% (w/v) sodium citrate solution (2.4 mL) was rapidly added, which was further boiled for 20 min and then cooled to 25 °C. The resultant product was diluted with 1% sodium citrate solution to obtain AuNP solution (0.1 nM) before they were grafted to the substrate. The distribution in the diameter of the AuNPs was characterized by dynamic light scattering analysis. AuNPs were conjugated to glass coverslip substrate. The glass substrate was cleaned with a mixture of HCl and methanol (1:1) for 30 min, activated with sulfuric acid for 1 h, and thiolated with mercaptopropylsilatrane (0.5 mM) in methanol for 1 h in the dark. The substrate was treated with AuNP solution (0.1 nM) for 15 min to obtain the AuNP-decorated substrate.

The surface of the AuNP on the substrate was coated with 0.2% (w/v) thiolated BP, including N,N-diisopropylethylamine (12 µL) and Tris(2-carboxyethyl)phosphine hydrochloride (TCEP) (10 mM), at 25 °C for 16 h in the dark, and then PEGylated. Thiolated PB was prepared by reacting 1% (w/v) pamidronate disodium (bisphosphonate, BP, Dalian Meilun Biology Technology) and 1% (w/v) thioglycolic acid with N-(3-dimethylaminopropyl)-N′-ethylcarbodiimide hydrochloride and N-hydroxysuccinimide in NaOH solution (pH = 8.0) at 25 °C for 16 h.

### The convertible formation of MgBNP or RGDBNP dimer

The BNP substrate was treated with pamidronate disodium (BP, 1 mM) and MgCl_2_ (1 mM) in tris-buffered saline (TBS, pH = 7.4) for 10 min to obtain MgBNP dimer. The MgBNP dimer was treated with EDTA (1 mM) in TBS for 10 min to dissolve Mg^2+^-BP NPs, which then reverts to the BNP substrate. The MgBNP substrate was treated with pamidronate disodium (BP, 0.8 mM), RGD-BP (0.2 mM), and MgCl_2_ (1 mM) in TBS for 10 min, to obtain RGDBNP dimer. The RGD-BP was prepared by conjugating thiolated RGD peptide (0.5 mM, GCGYGRGDSPG, GenScript) to acryloyl BP (2 mM) with TCEP (5 mM) at 37 °C for 16 h. The acryloyl BP was prepared by reacting 1% (w/v) pamidronate disodium (BP) and 1% (w/v) N-acryloxysuccinimide in NaOH solution at 25 °C for 16 h.

Transmission electron microscope (TEM) imaging (CM-200, Philips) and high-resolution TEM imaging (JEOL JEM-ARM200F) were carried out to characterize the convertible dimer formation using TEM grid (Dune Sciences). The approximate diameter and density of the Mg^2+^-BP NPs was determined with the Image J software. Energy dispersive spectroscopy analysis was performed to characterize the convertible dimer formation for gold (Au) element in the AuNPs as well as magnesium (Mg) and phosphorous (P) element in the Mg^2+^-BP NPs. The binding of integrin β1 to the substrate was analyzed by incubating the substrate with integrin β1 (50 µg/mL) at 37 °C for 30 min and staining for integrin β1 (1:100, sc-9970, Santa Cruz Biotechnology).

### Convertible nanoassembly-controlled macrophage attachment

Macrophages (RAW 264.7 from ATCC, TIB-71) at passage 5 were plated onto the BNP substrate at a density of 50,000 cells/cm^2^ and cultured in basal medium [high-glucose DMEM with 10% (v/v) heat-inactivated fetal bovine serum and penicillin/streptomycin] at 37 °C and 5% CO_2_. Cells were treated in the identical conditions used for MgBNP formation, reversion to BNP and RGDBNP formation, and then replaced with basal medium. The real-time fluorescent confocal microscopy imaging was performed for the cells stained with calcein AM. The viability of the macrophages was determined for the cells stained with calcein AM and propidium iodide.

### Dimer formation-regulated macrophage polarization

Macrophages were cultured under M1-type-polarizing medium [basal medium containing 10 ng/mL lipopolysaccharide (LPS) and 10 ng/mL recombinant interferon-gamma (IFN-γ)] or M2-type-polarizing medium [basal medium containing 20 ng/mL interleukin-4 (IL-4) and 20 ng/mL interleukin-13 (IL-13)] with or without Y27632 (50 µM, Abcam). The MgBNP or RGDBNP dimer was formed at the beginning of culture.

To quantify secreted levels of cytokines by macrophages, enzyme-linked immunosorbent assay (ELISA) was performed by using the ELISA plate, HRP-conjugate reagent, and chromogen solution for absorbance reading at 450 nm. Flow cytometry was performed with BD LSR Fortessa for iNOS (1:100, sc-7271, Santa Cruz Biotechnology) and Arg-1 (1:100, NBP2-03618, Novus Biologicals) and analyzed with FlowJo software. The fusion of macrophages was evaluated after their culture under a medium containing IL-4 and IL-13, stimulators for macrophage fusion. The macrophages were analyzed by immunofluorescent staining. Cells were treated with 4% (w/v) paraformaldehyde and 0.25% (v/v) Triton-X for 10 min and then blocked with 3% (w/v) bovine serum albumin for 30 min. Cells were incubated in primary antibodies against vinculin (1:400, v9131, Sigma-Aldrich), iNOS (1:100, sc-7271, Santa Cruz Biotechnology), Arg-1 (1:100, ab91279, Abcam), ROCK2 (1:100, ab71598, Abcam), or NIMP-R14 (1:100, sc-59338, Santa Cruz Biotechnology) at 4 °C for 16 h. Cells were treated with secondary antibodies (1:250, Thermo Scientific), phalloidin (1:100, Molecular Probes), or DAPI (1:1000, Molecular Probes) at 25 °C for 45 min. Cell were imaged using confocal microscope (Nikon) and then analyzed with Image J software. Western blotting was performed with ChemiDoc Touch Imaging System (Bio-Rad) for iNOS (1:400, sc-7271, Santa Cruz Biotechnology), Arg-1 (1:400, sc-271430, Santa Cruz Biotechnology), or GAPDH (1:800, sc-365062, Santa Cruz Biotechnology) and presented with molecular weight markers, which were subjected to densitometric analysis using Image J software. Reverse transcription-quantitative polymerase chain reaction (RT-qPCR) was performed to analyze expression levels of iNOS, CD80, Arg-1, or Ym2 gene after normalization to GAPDH gene, by using TRIzol, RevertAid First Strand cDNA Synthesis Kit and TaqMan assays.

### Dimer formation-regulated polarization of host macrophages

Forty, 3-month-old male BALB/c mice were used with five mice per group in compliance with all relevant ethical regulations after the approval of the Institutional Animal Care and Use Committee at the Chinese University of Hong Kong. The mice were anesthetized by the intraperitoneal injection of ketamine (100 mg/kg) and xylazine (10 mg/kg). A 2-cm-long incision was made in the back of each mouse. The BNP silicon substrate was implanted into the subcutaneous pocket, and the skin was closed. BP (1.2 µg) and Mg^2+^ (0.1 µg) were administered to form MgBNP dimer. EDTA (1.5 µg) was then administered to revert to the BNP. BP (1 µg), RGD-BP (0.2 µg), and Mg^2+^ (0.1 µg) were administered to form RGDBNP dimer. IL-4 (100 ng) and IL-13 (100 ng) were administered as M2 stimulators. The mice were housed in the cages and sacrificed at 24 h post implantation. To examine maturation of host macrophages to fuse and thus form foreign body giant cells, PEGDA-based hydrogels containing thiol group were used for AuNP coupling to yield the BNP substrate, which was imaged by scanning electron microscopy. The BNP substrate was subcutaneously implanted and subjected to form the MgBNP or RGDBNP dimer in situ, which then received M2 stimulators. The substrates were analyzed at 10 days post implantation by paraffin embedding and immunohistochemical staining for F4/80 (1:100, sc-377009, Santa Cruz Biotechnology) with methyl green counterstaining (Abcam).

### Statistical analyses

All the experiments in this study were repeated at least two times independently and analyzed with Graphpad Prism 5.00 software. Statistical analyses were carried out with two-tailed Student’s *t*-test or ANOVA, and *p*-values <0.05 were considered statistically significant differences between the compared groups, to which different asterisks were assigned (*p < 0.05; **p < 0.01; ***p < 0.001).

### Reporting Summary

Further information on experimental design is available in the Nature Research Reporting Summary linked to this article.

## Supplementary information

Supplementary Information

Description of Additional Supplementary Files

Supplementary Movie 1

Reporting Summary

Source Data

## Data Availability

All data are available from the authors on reasonable request. The source data underlying some of Figs. and Supplementary Figs. are provided as a Source Data file.
