# Supplementary material for: Immunoregulation of macrophages by dynamic ligand presentation via ligand–cation coordination
Source: Nat Commun. 2019 Apr 12;10:1696. doi: 10.1038/s41467-019-09733-6 (PMC6461616; doi:10.1038/s41467-019-09733-6)
Supplement: Supplementary file 1 — Supplementary Information [file 41467_2019_9733_MOESM1_ESM.pdf]

Supplementary Information

Immunoregulation of macrophages by dynamic ligand presentation via ligand–cation coordination

Kang et al.

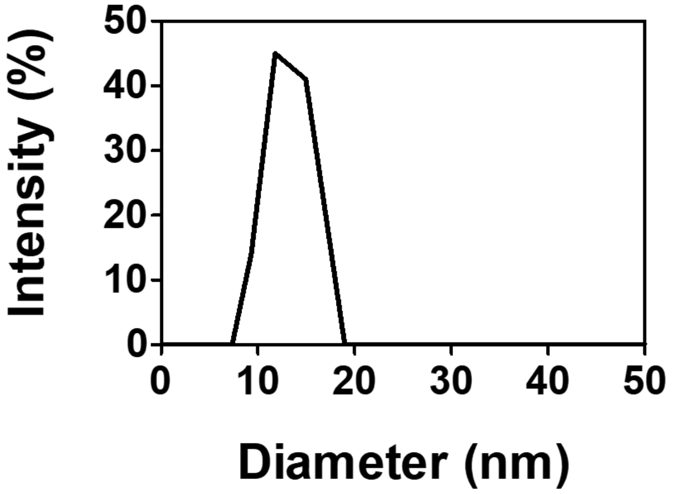

Supplementary Fig. 1 Characterization of the gold nanoparticle (AuNP). Dynamic light scattering analysis of the distribution in the diameter of the AuNPs.

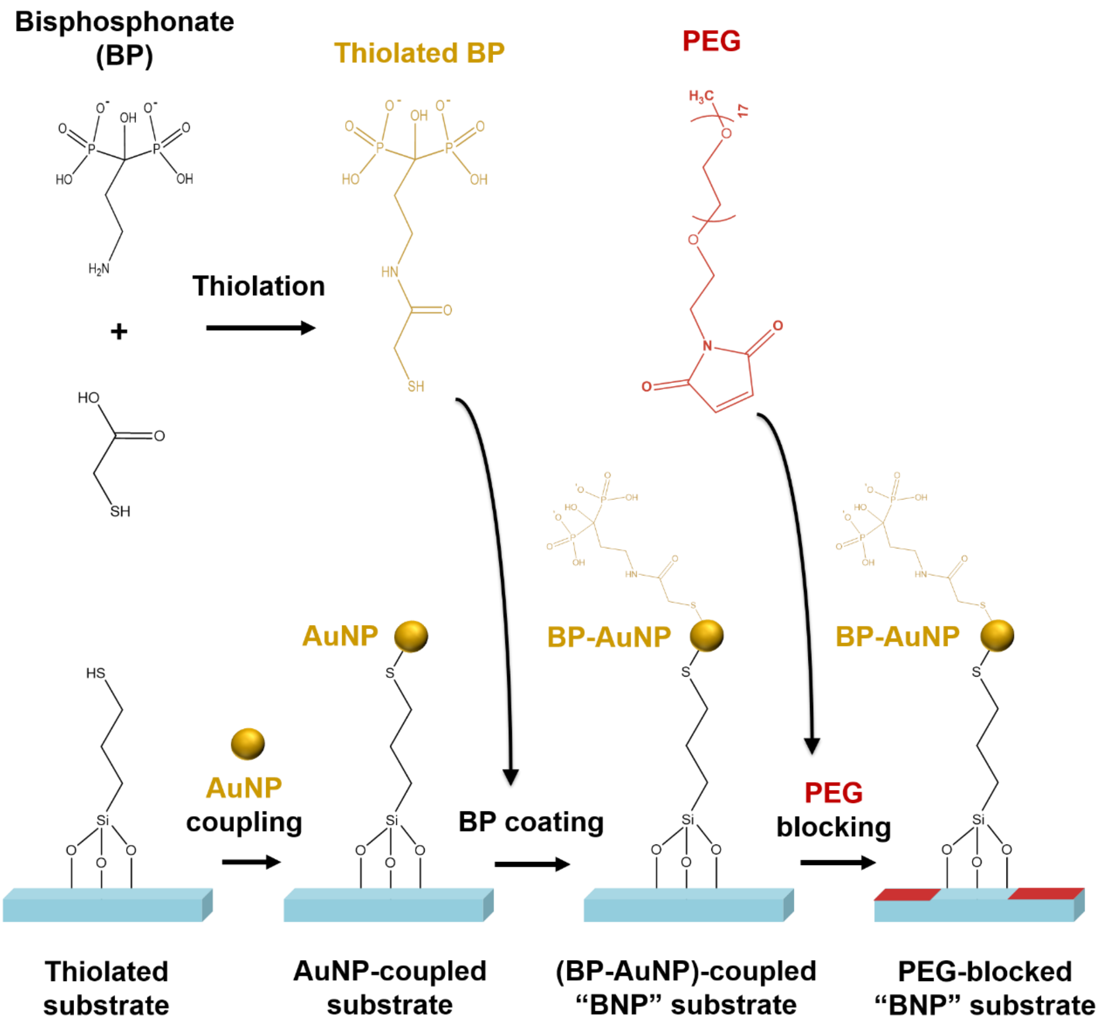

Supplementary Fig. 2 Summary of the experimental procedures used to couple the BP-bearing AuNP monomer (BNP) to the substrate. Schematic representation of the serial coupling of the gold nanoparticle (AuNP) to the thiolated glass substrate through gold-sulfur bonding, and then the coupling of thiolated bisphosphonate (BP) to the AuNP on the substrate through gold-sulfur bonding. The BP coating on the surface of AuNP allowed the reversible nanoassembly of Mg^{2+}-monofunctional Mg^{2+}-BP nanoparticle on the BP-AuNP *via* reversible Mg^{2+}-BP coordination. The BP-AuNP-coupled substrate was subsequently passivated by PEGylation on the remaining area of the substrate that was not covered with the BP-AuNP, to minimize the non-specific adhesion of macrophages.

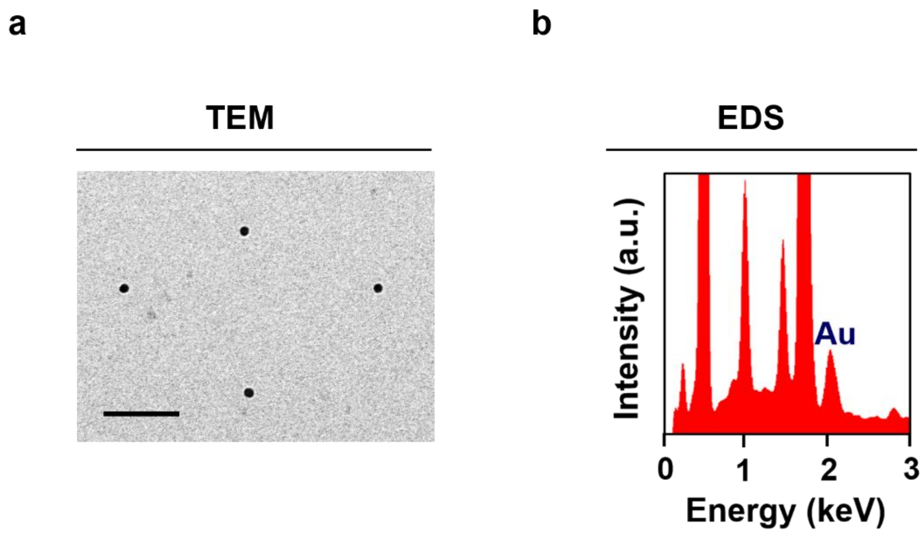

Supplementary Fig. 3 Characterization of the substrate coupling of the BP-bearing AuNP monomer template (BNP). **a** Transmission electron micrograph of the BP-AuNP-conjugated substrate. Scale bar is 100 nm. **b** Energy dispersive spectrum of the BP-AuNP-grafted substrate. Detection of elemental Au confirmed the successful grafting of the AuNPs to the substrate.

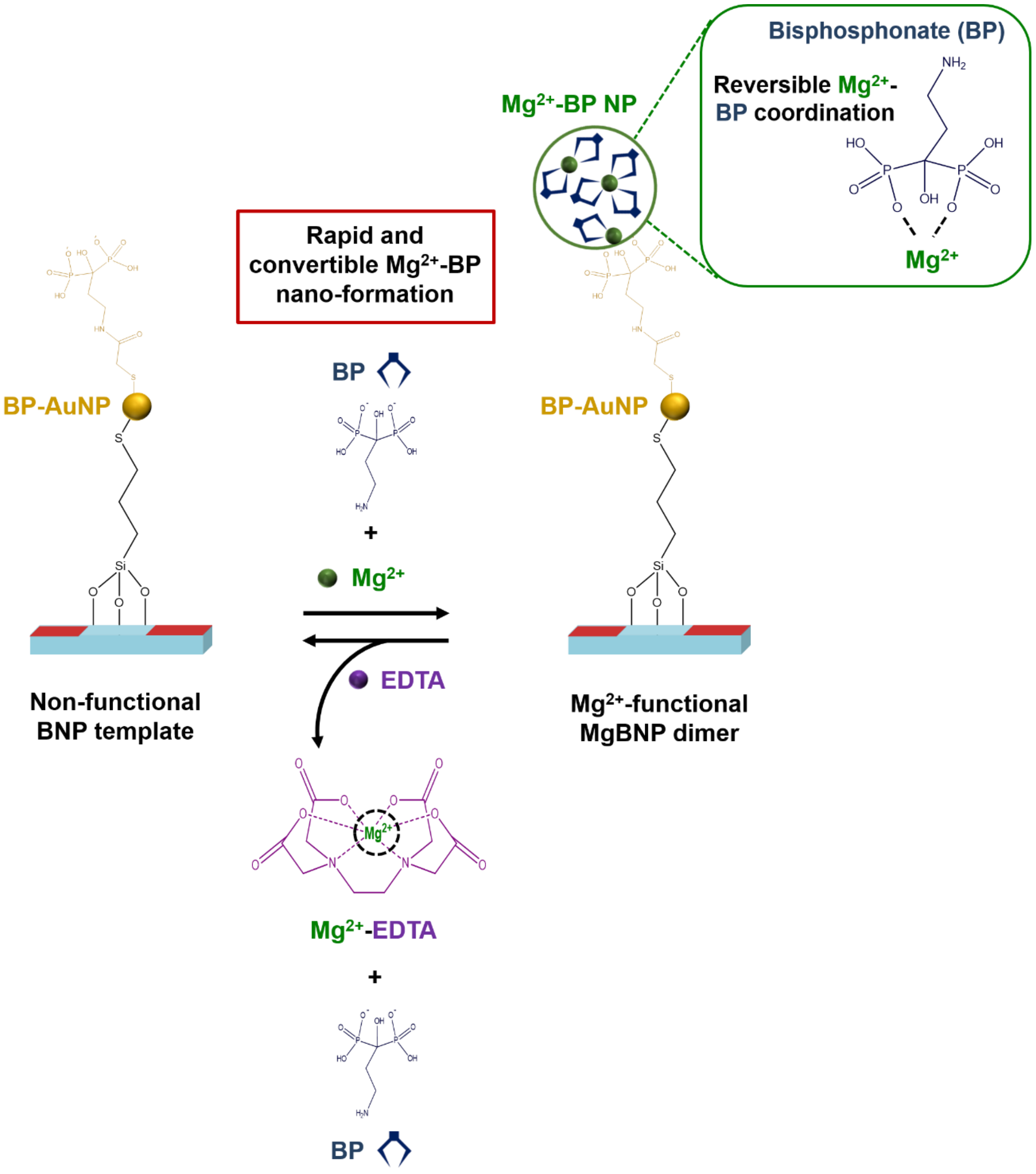

Supplementary Fig. 4 Summary of the chemical reactions of the reversible nanoassembly. Mg^{2+} and BP rapidly induced the nanoassembly of cell-adhesive Mg^{2+}-monofunctional Mg^{2+}-BP NP into the (Mg^{2+}-BP)-Au dimer (MgBNP). EDTA rapidly induced the nano-disassembly of the Mg^{2+}-BP NP into the non-adhesive “BNP” template. The reversibility of the Mg^{2+}-BP coordination allowed this reversible nanoassembly and nano-disassembly.

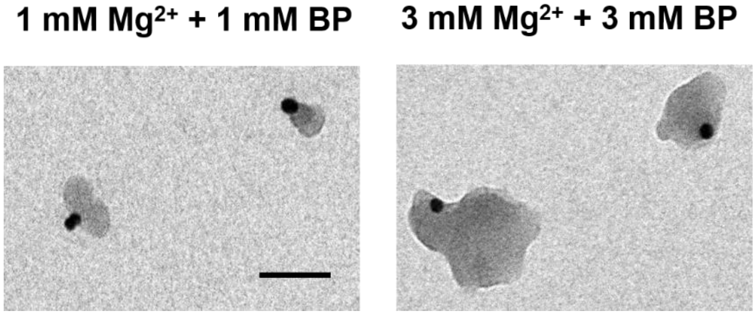

Supplementary Fig. 5 Transmission electron micrograph of the grown Mg^{2+}-BP NPs on the (BP-AuNP)-substrate in the presence of various concentrations of Mg^{2+} and BP. Scale bar is 50 nm.

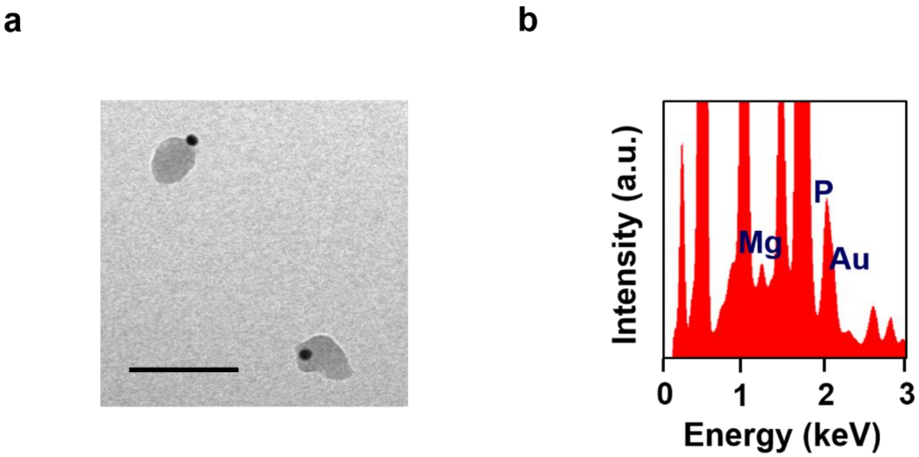

Supplementary Fig. 6 The nanoassembled Mg^{2+}-BP NPs in the MgBNP dimers are stable under culture conditions. **a** Transmission electron microscopy image and **b** energy dispersive spectroscopy of the MgBNP dimers after their incubation in basal culture medium at 37 ^{o}C for 7 d. Scale bar is 100 nm. Au element indicates the AuNPs on the substrate, and elemental Mg and P confirm the nanoassembled Mg^{2+}-BP NPs.

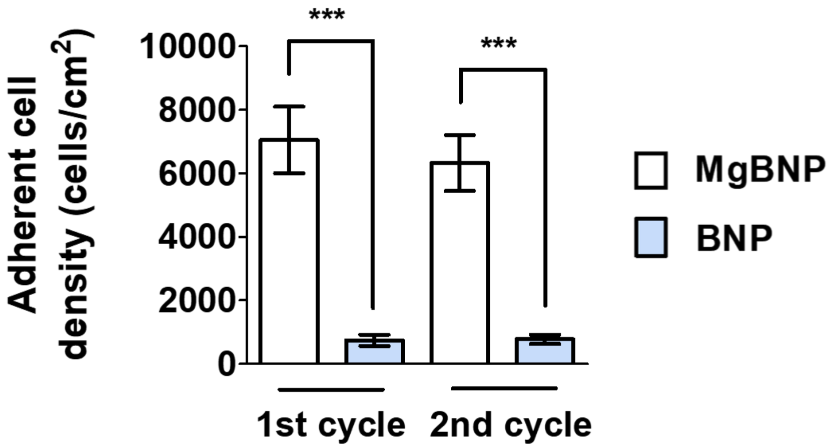

Supplementary Fig. 7 Rapid and reversible nanoassembly allows the cyclic attachment and detachment of macrophages through reversible Mg^{2+}-BP coordination. Quantification of adherent macrophages after their cyclic adhesion to the nanoassembled MgBNP and detachment from the nano-disassembled BNP template (shown in Fig. 1b). Macrophages adhered to the dimeric substrate for 12 h after the nanoassembly for 10 min. Macrophages were detached during the nano-disassembly for 10 min. Data are means ± s.d. (n=4). ***P < 0.001 (two-tailed Student's t-test).

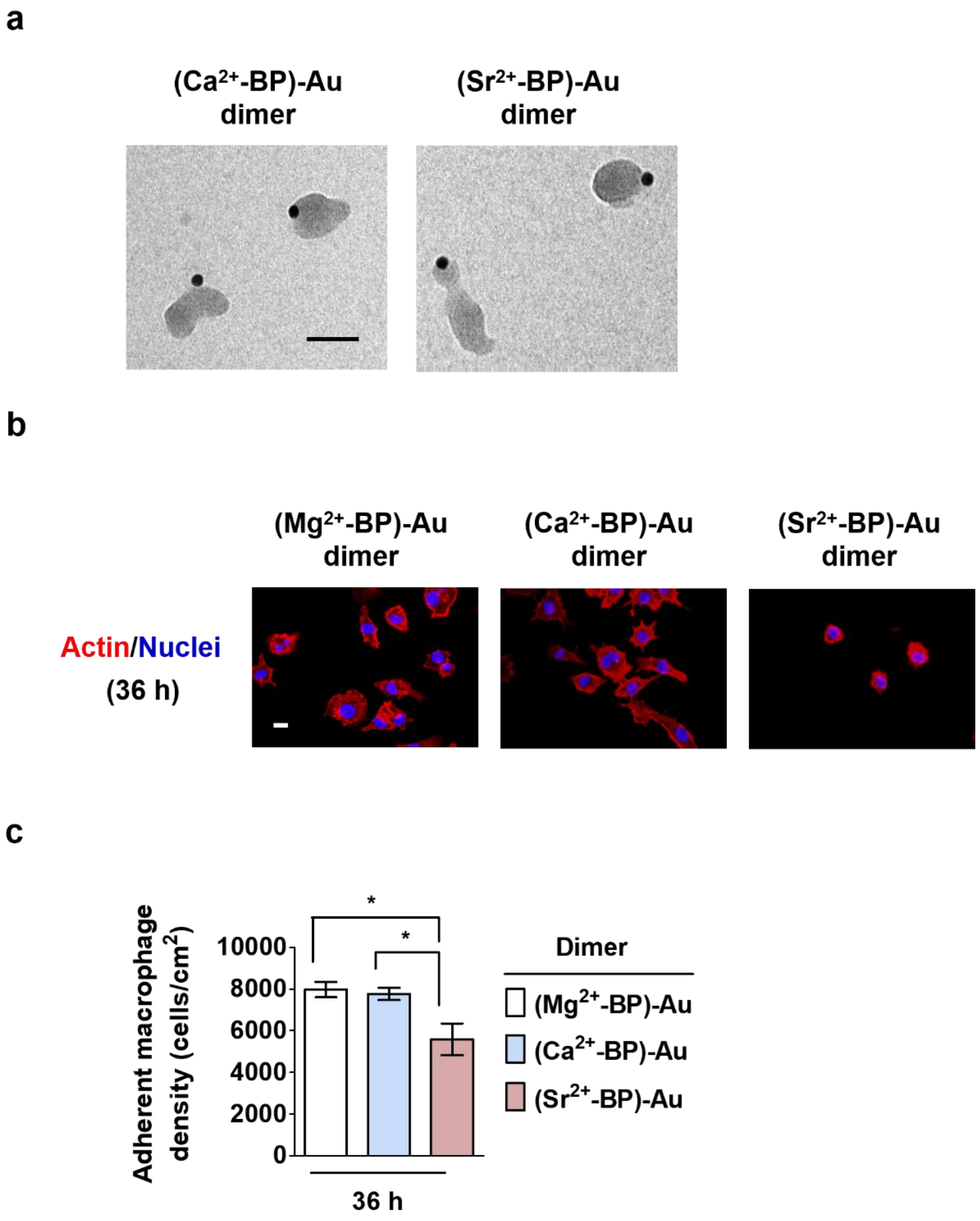

Supplementary Fig. 8 Metal ion-dependent adhesion of macrophages to the dimeric substrate. **a** Transmission electron microscopy image of the grown Ca^{2+}-BP NPs or Sr^{2+}-BP NPs on the BP-coated AuNP (BNP) substrate. Scale bar is 50 nm. **b** Fluorescent images of macrophages stained for actin (in red) and nuclei (in blue), which adhered to the (Mg^{2+}-BP)-Au, (Ca^{2+}-BP)-Au, or (Sr^{2+}-BP)-Au dimeric substrate, and **c** corresponding quantification of adherent macrophage density. Scale bar is 50 µm. Data are means ± s.e.m. (n=4). *P < 0.05 (ANOVA).

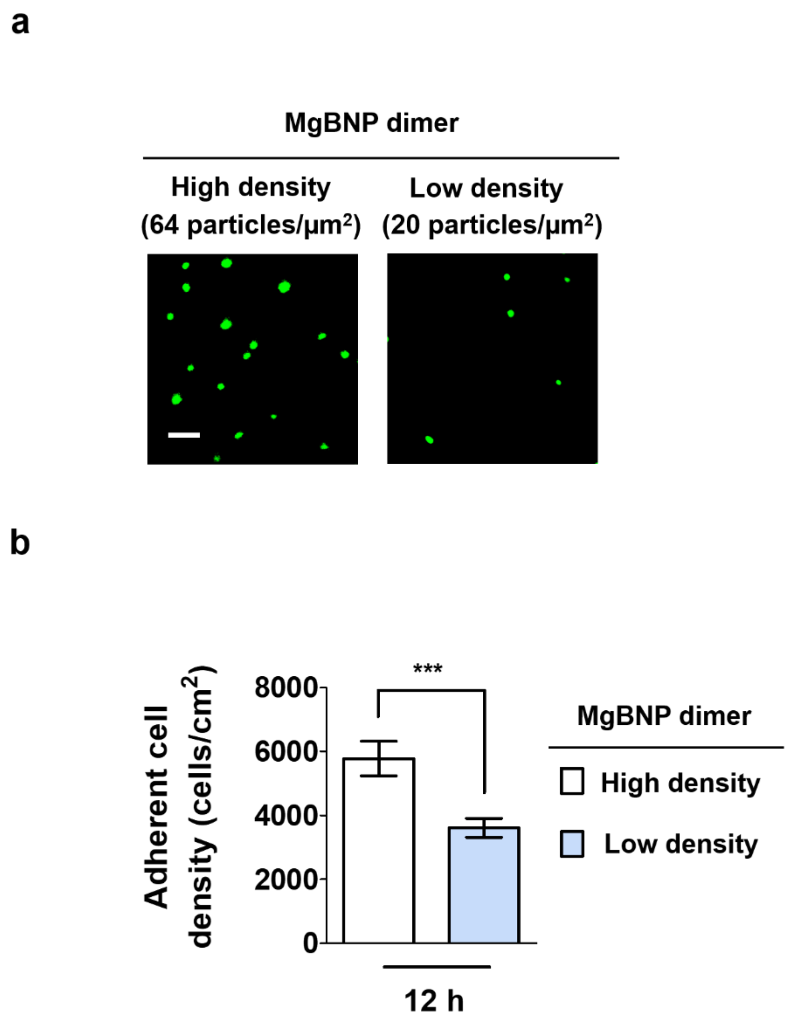

Supplementary Fig. 9 Dimer density-dependent adhesion of macrophages to the MgBNP dimeric substrate. **a** Fluorescently stained images of macrophages adhered to the MgBNP dimeric substrate with high dimer density (64 ± 19 particles/μm^2) and low dimer density (20 ± 6 particles/μm^2), and **b** corresponding quantification of adherent macrophage densities. Scale bar is 100 μm. Data are means ± s.d. (n=4). ***$P < 0.001$ (two-tailed Student's t-test).

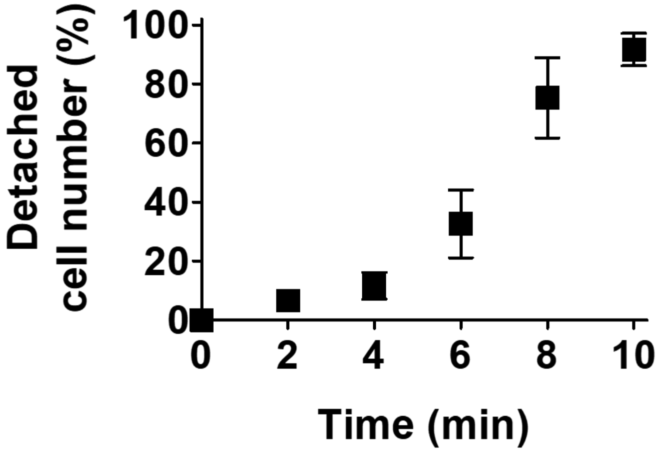

Supplementary Fig. 10 The nano-disassembly mediates the rapid detachment of macrophages during 10 min of treatment of the MgBNP dimeric substrate with EDTA. Real-time quantification of the detached macrophages after treatment with EDTA during 10 min, relative to the initially attached macrophages before treatment (shown in Fig. 1d). Data are means ± s.d. (n=3).

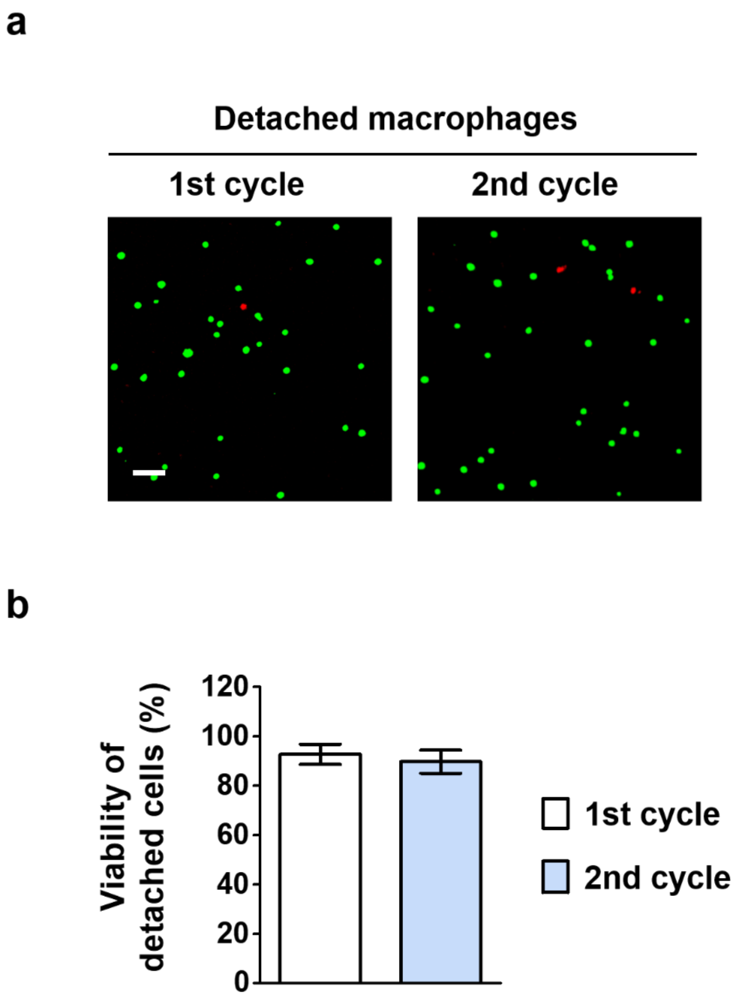

Supplementary Fig. 11 Viable macrophages after two successive cycles of attachment and detachment by the reversible nanoassembly. **a** Fluorescently stained images of live (in green) and dead (in red) macrophages in suspension immediately after they were detached in two successive cycles. **b** The corresponding quantification of the viability of the macrophages detached in the two successive cycles. Scale bar is 100 μm. The viability of the macrophages was determined with a live-dead assay as the percentage of viable cells after the cells were exposed to the nanoassembly for 10 min, to subsequently allow them to adhere to the MgBNP dimeric substrate for 12 h, and then detached by the nano-disassembly for 10 min. Data are means ± s.d. (n=3).

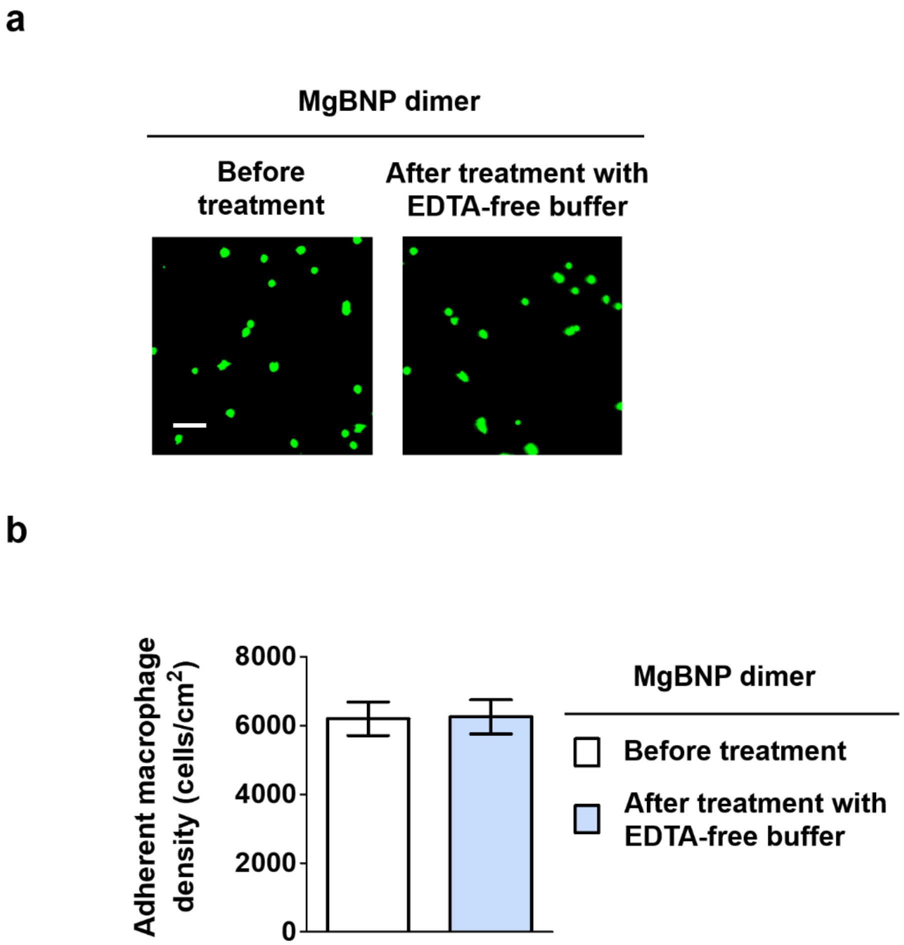

Supplementary Fig. 12 EDTA is vital in mediating the detachment of macrophages adherent to the MgBNP dimeric substrate. **a** Fluorescently stained images of adherent macrophages, and **b** corresponding quantification of adherent macrophage densities, before and after treatment with EDTA-free buffer. Scale bar is 100 µm. Data are means ± s.d. (n=4).

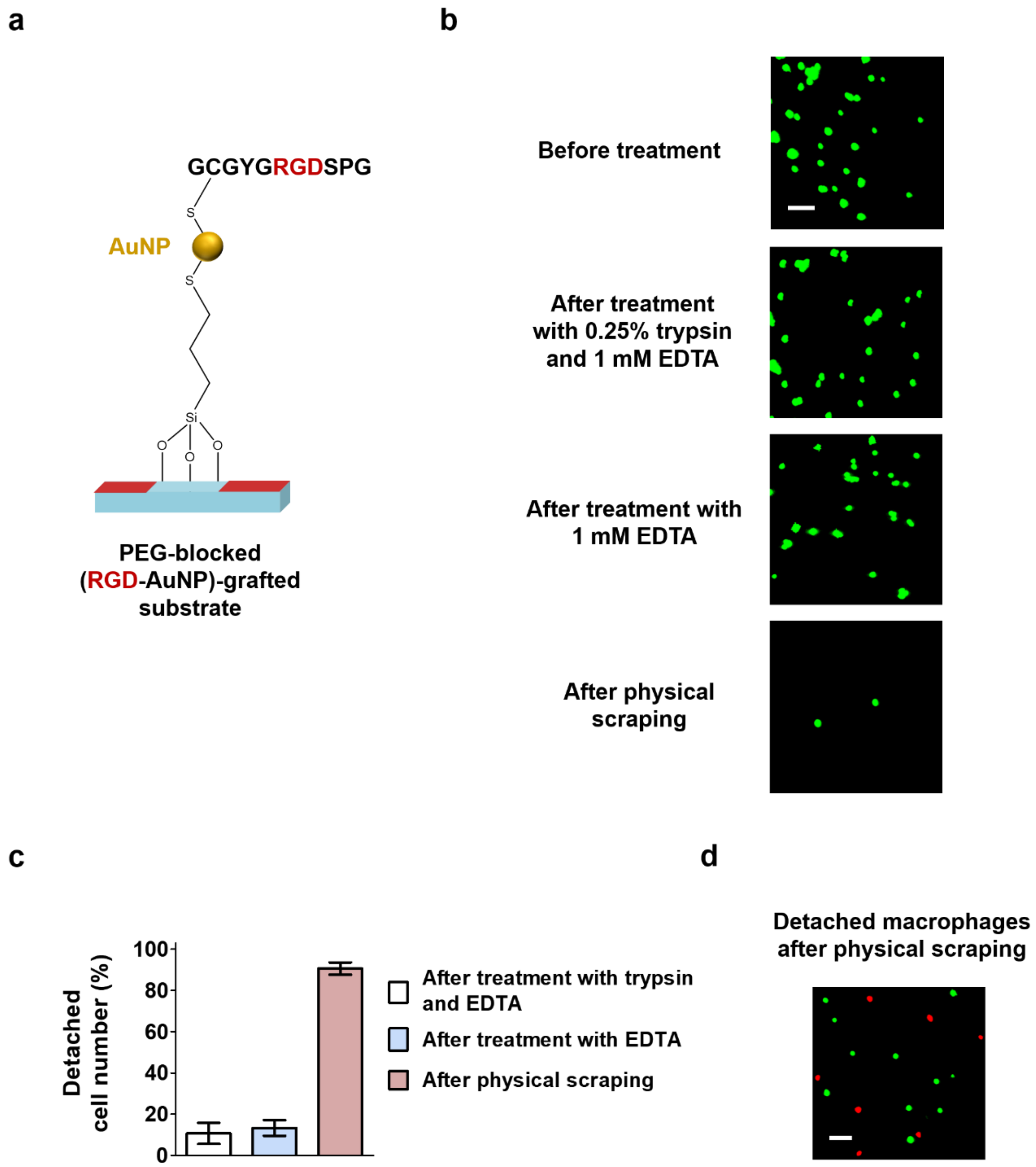

Supplementary Fig. 13 Release of macrophages from RGD-AuNP-coated substrate by trypsin-EDTA, EDTA, or physical scraping. **a** Schematic representation of the RGD-AuNP-coated substrate. **b** Fluorescently stained images of adherent macrophages before and after trypsin-EDTA treatment, EDTA treatment, or physical scraping of the substrate, and **c** corresponding quantification of the detached cell number. **d** Fluorescently stained images of live (in green) and dead (in red) macrophages in suspension immediately after they were detached by physical scraping of the substrate. Scale bar is 100 μm. Data are means ± s.e.m. (n=3).

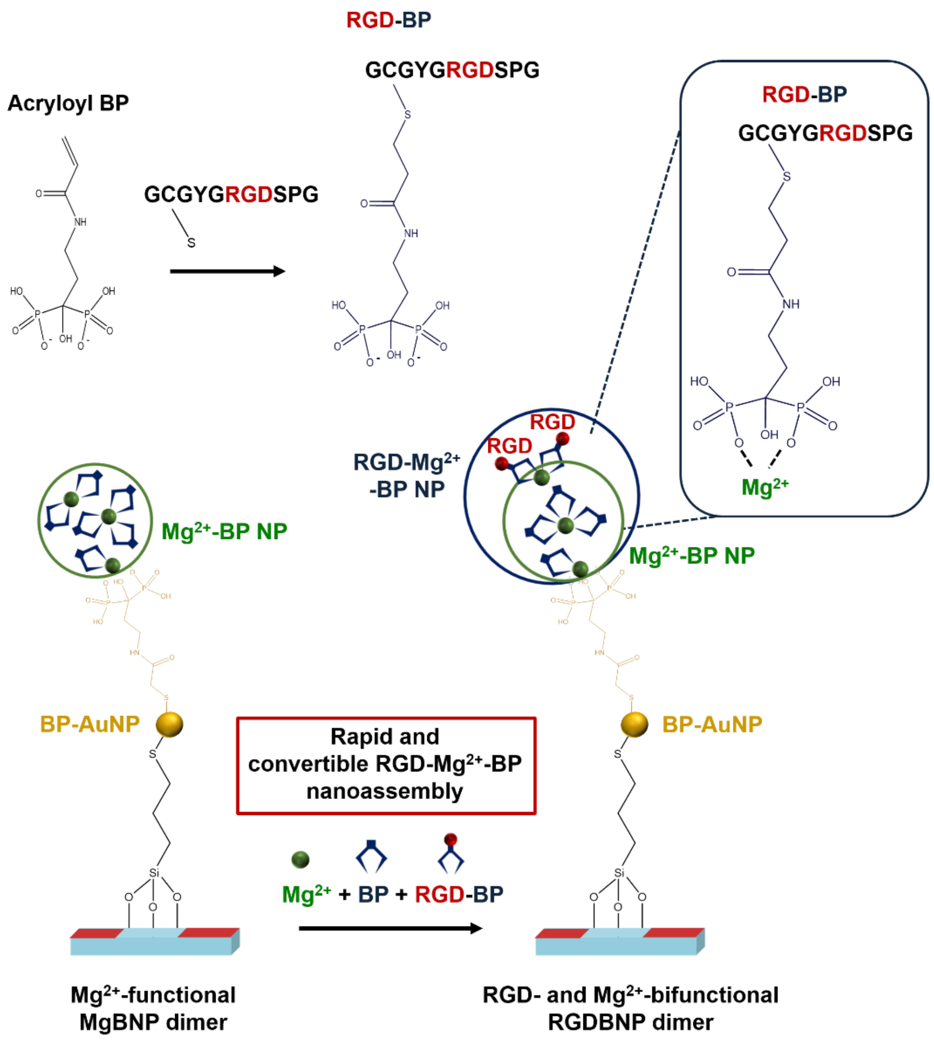

Supplementary Fig. 14 Schematic representation of the biofunctional dimeric nanoassembly. RGD-coupled BP, BP, and Mg^{2+} rapidly induced the nanoassembly of RGD- and Mg^{2+}-bifunctional RGD-Mg^{2+}-BP nanoparticles on the (Mg^{2+}-BP)-Au dimer (MgBNP) to form the (RGD-Mg^{2+}-BP)-Au dimer (RGDBNP).

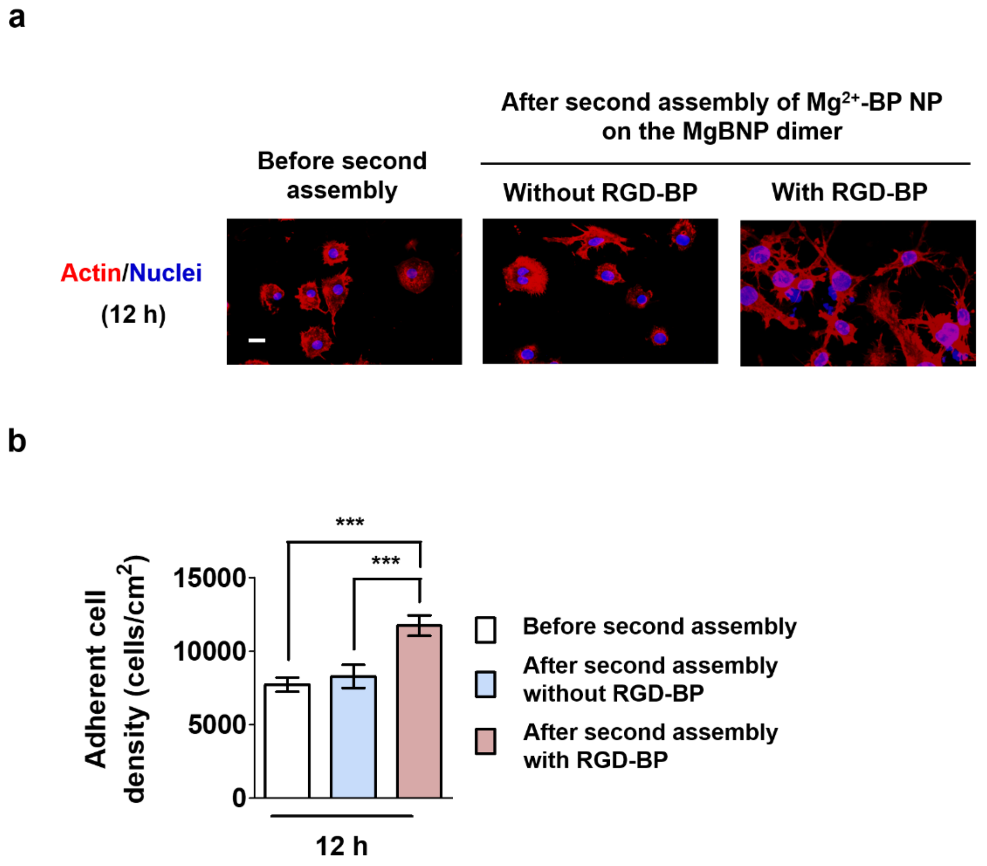

Supplementary Fig. 15 RGD-BP is required in the second assembly of the Mg^{2+}-BP NPs on the (Mg^{2+}-BP)-Au dimer (MgBNP) to significantly enhance macrophage adhesion. **a** Fluorescently stained images of adherent macrophages for actin (in red) and nuclei (in blue) before or after the second assembly of Mg^{2+}-BP NPs on the MgBNP dimer with and without RGD-BP. Scale bar is 50 µm. **b** Corresponding quantification of adherent macrophage densities. Data are means ± s.d. (n=4). ***P < 0.001 (ANOVA).

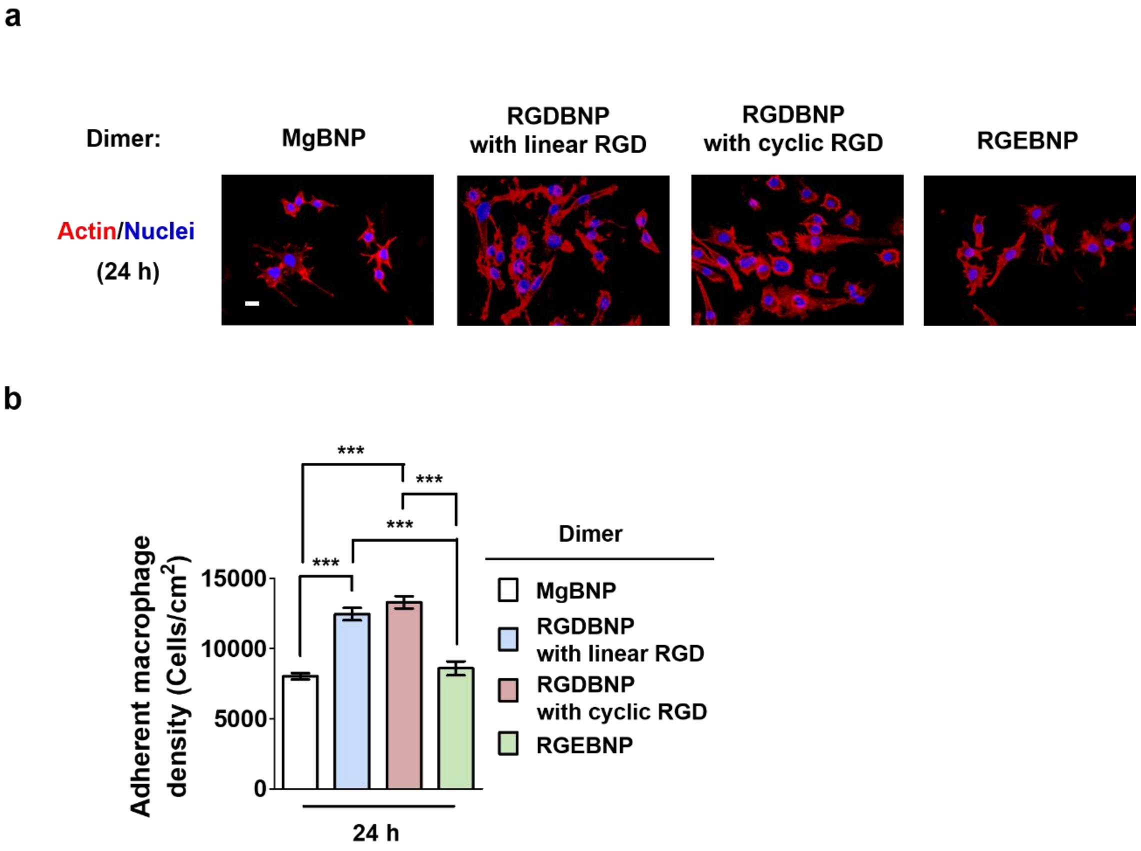

Supplementary Fig. 16 RGD-BP included in the in the second assembly of the Mg^{2+}-BP NPs is functional in promoting macrophage adhesion. **a** Fluorescently stained images of macrophages for actin (in red) and nuclei (in blue), which adhered to the dimeric substrates with MgBNP, RGDBNP with linear RGD peptides, RGDBNP with cyclic RGD peptides, or RGEBNP with RGE peptide (mutated RGD), and **b** corresponding quantification of adherent macrophage density. Scale bar is 50 µm. Data are means ± s.e.m. (n=4). ***$P < 0.001$ (ANOVA).

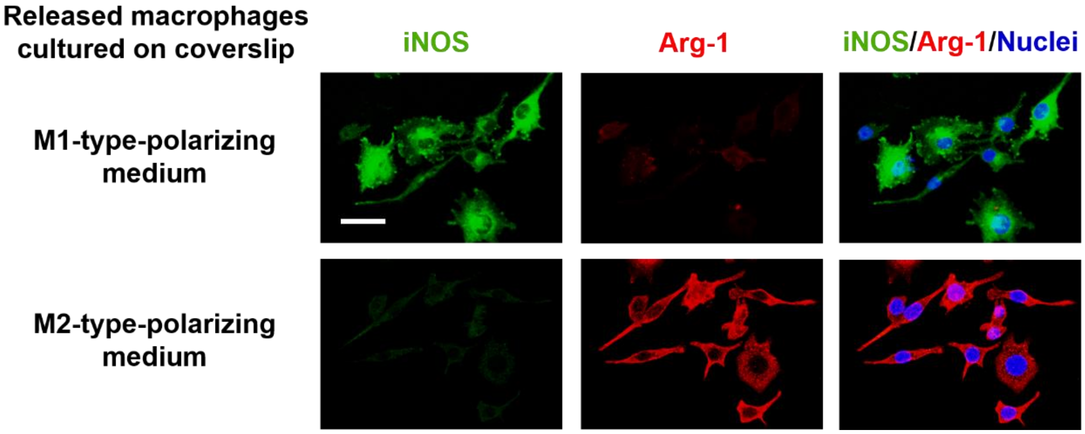

Supplementary Fig. 17 Macrophages maintain their polarization capacity after their detachment *via* the dissolution of Mg^{2+}-BP NPs from the MgBNP substrate. Fluorescent images of macrophages immunostained for iNOS (in green), Arg-1 (in red), and nuclei (in blue) at 36 h after culture (the first 12 h under culture in basal medium, followed by 24 h under culture in M1-type- or M2-type-polarizing medium). Macrophages re-adhered to a coverslip after they were detached *via* EDTA-mediated disassembly of Mg^{2+}-BP NPs for 10 min.

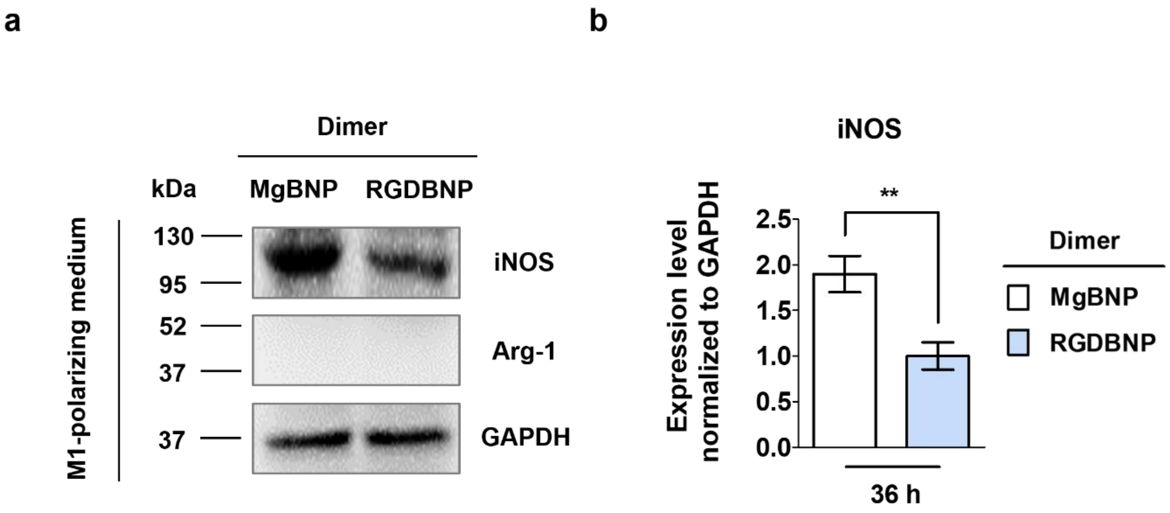

Supplementary Fig. 18 Western blotting analysis of the suppression of M1-type polarization of macrophages by the dimeric nanoassembly presenting RGD-BP. **a** Western blotting image of iNOS, Arg-1, or GADPH, and **b** corresponding quantification of the intensities of iNOS expression in macrophages after 36 h under culture (the first 12 h under culture in basal medium, followed by 24 h under culture in M1-type-polarizing medium. The dimeric nanoassembly of Mg^{2+}-BP NPs (MgBNP dimer) or RGD-Mg^{2+}-BP NPs (RGDBNP dimer) was induced after 0 h under culture. The intensity of iNOS expression is presented as its relative expression after normalization to that of GADPH, and then to that in the RGDBNP dimer. Data are means ± s.d. (n=3). **$P < 0.01$ (two-tailed Student’s t-test).

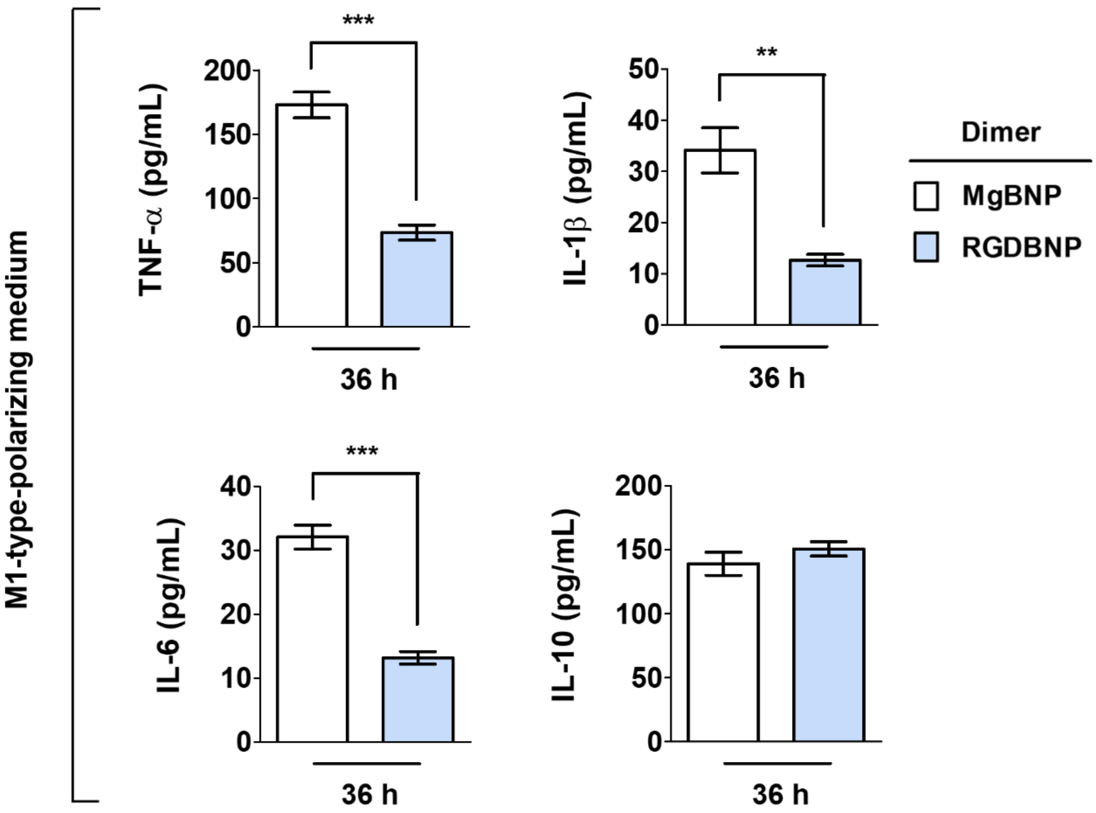

Supplementary Fig. 19 The dimeric nanoassembly bearing RGD-BP inhibits the secretion of pro-inflammatory cytokines. Levels of secreted pro-inflammatory cytokines, such as TNF-α, IL-1β, and IL-6, or anti-inflammatory cytokines, such as IL-10, from adherent macrophages in the MgBNP dimer or RGDBNP dimer after 36 h under culture in the M1-type-polarizing medium. Data are means ± s.d. (n=3). **P < 0.01, ***P < 0.001 (two-tailed Student's t-test).

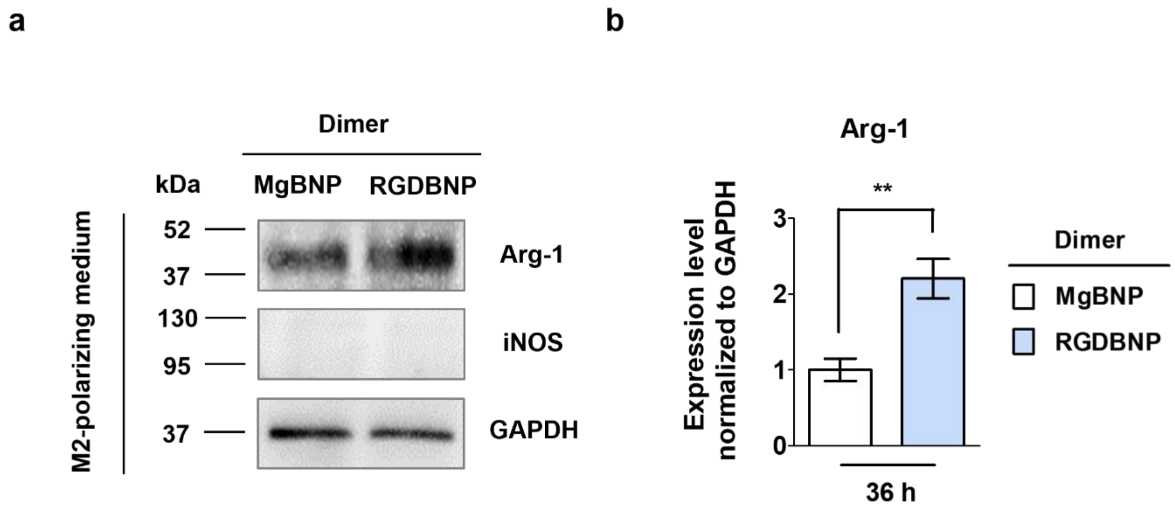

Supplementary Fig. 20 Western blotting analysis of the stimulation of M2-type polarization of macrophages by the dimeric nanoassembly bearing RGD-BP. **a** Western blotting image of Arg-1, iNOS, or GADPH and **b** corresponding quantification of the intensities of Arg-1 expression in macrophages after 36 h under culture (the first 12 h under culture in basal medium, followed by 24 h under culture in M2-type-polarizing medium). The dimeric nanoassembly of Mg^{2+}-BP-NPs (MgBNP dimer) or the RGD-Mg^{2+}-BP NPs (RGDBNP dimer) was directed after 0 h under culture. The intensity of Arg-1 expression is presented as its relative expression after normalization to that of GADPH, and then to that in the MgBNP dimer. Data are means ± s.d. (n=3). **P < 0.01 (two-tailed Student's t-test).

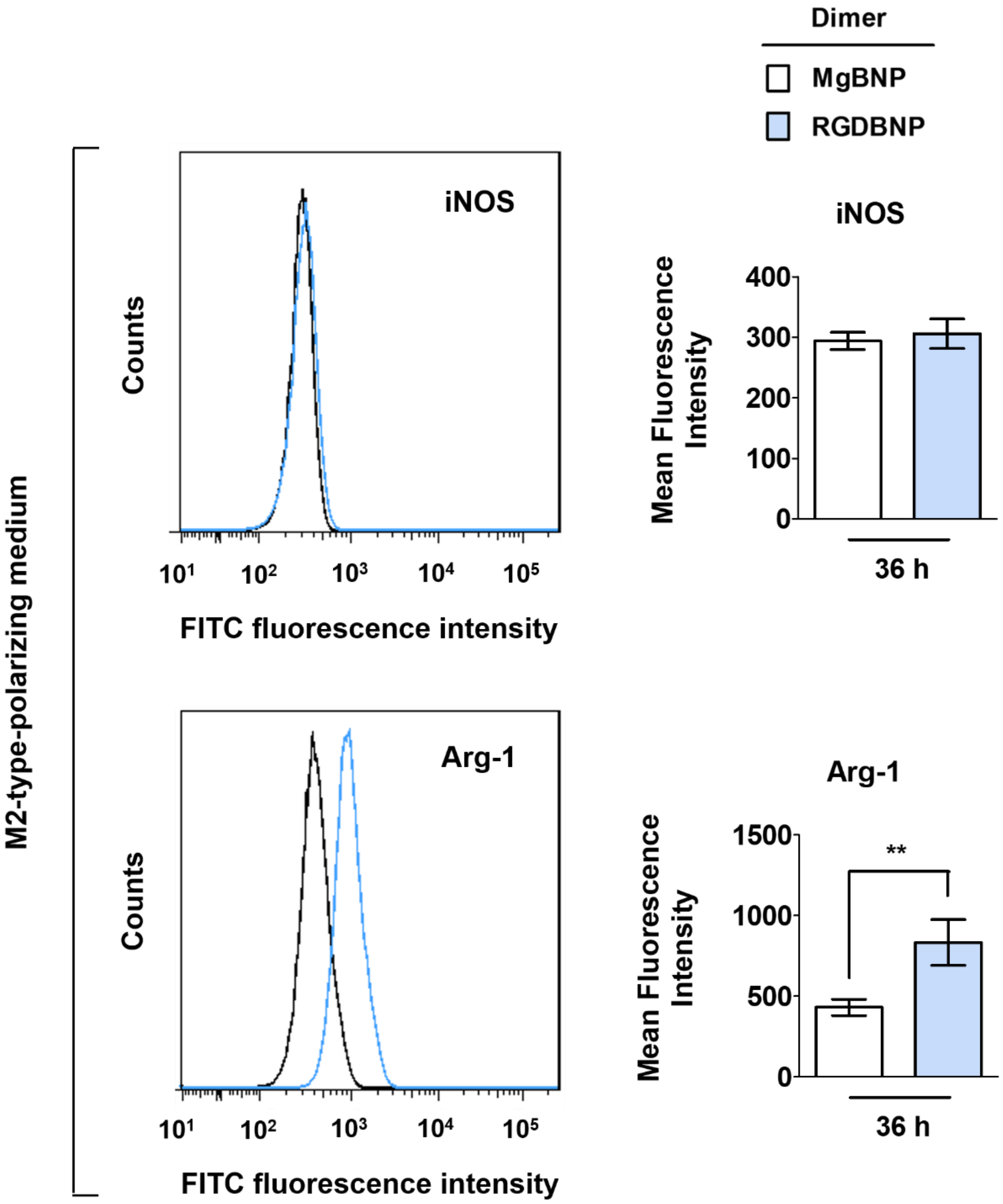

Supplementary Fig. 21 Flow cytometry measurements confirm the RGD-presenting dimer-stimulated expression of M2-type marker. Representative flow cytometry histograms (Left) and corresponding mean fluorescence intensity (Right) of iNOS or Arg-1 for adherent macrophages in the MgBNP dimer or RGDBNP dimer after 36 h under culture in the M2-type-polarizing medium. Data are means ± s.d. (n=3). **P < 0.01 (two-tailed Student's t-test).

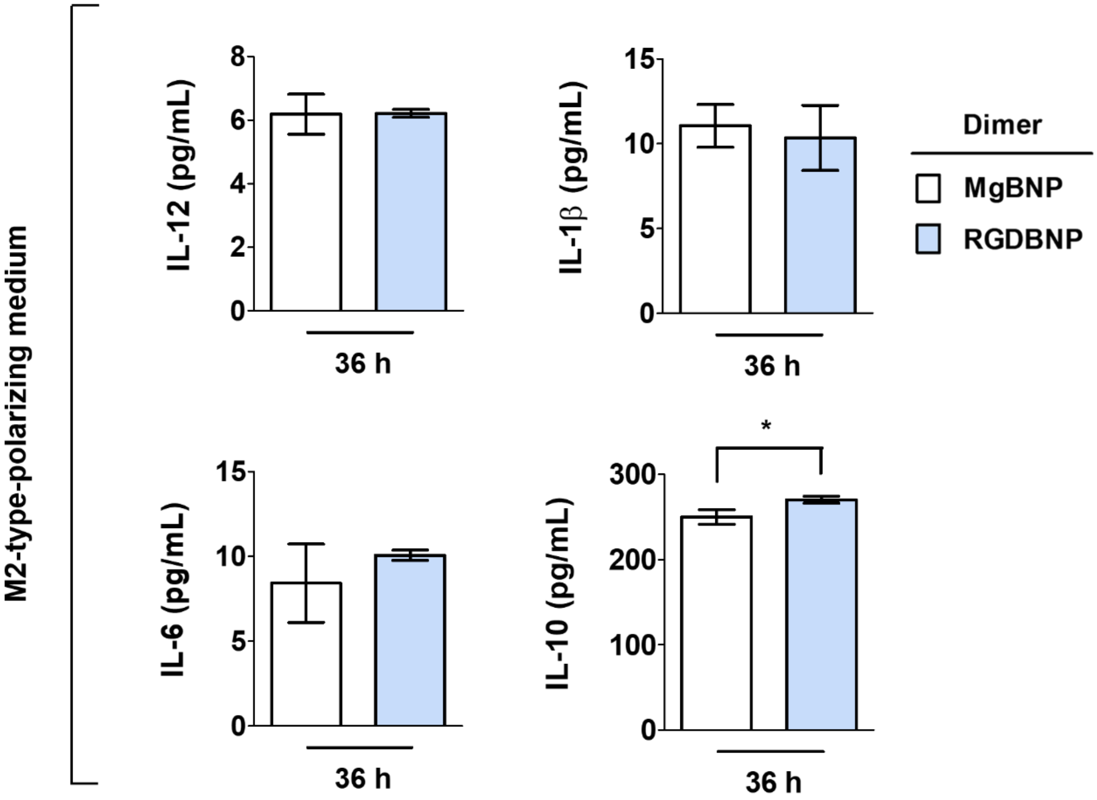

Supplementary Fig. 22 The dimeric nanoassembly presenting RGD-BP promotes the secretion of anti-inflammatory cytokines. Levels of secreted anti-inflammatory cytokines, such as IL-10, or pro-inflammatory cytokines, such as IL-12, IL-1β, and IL-6, from adherent macrophages in the MgBNP or RGDBNP dimer after 36 h under culture in the M2-type-polarizing medium. Data are means ± s.d. (n=3). $^{*}P < 0.05$ (two-tailed Student's t-test).

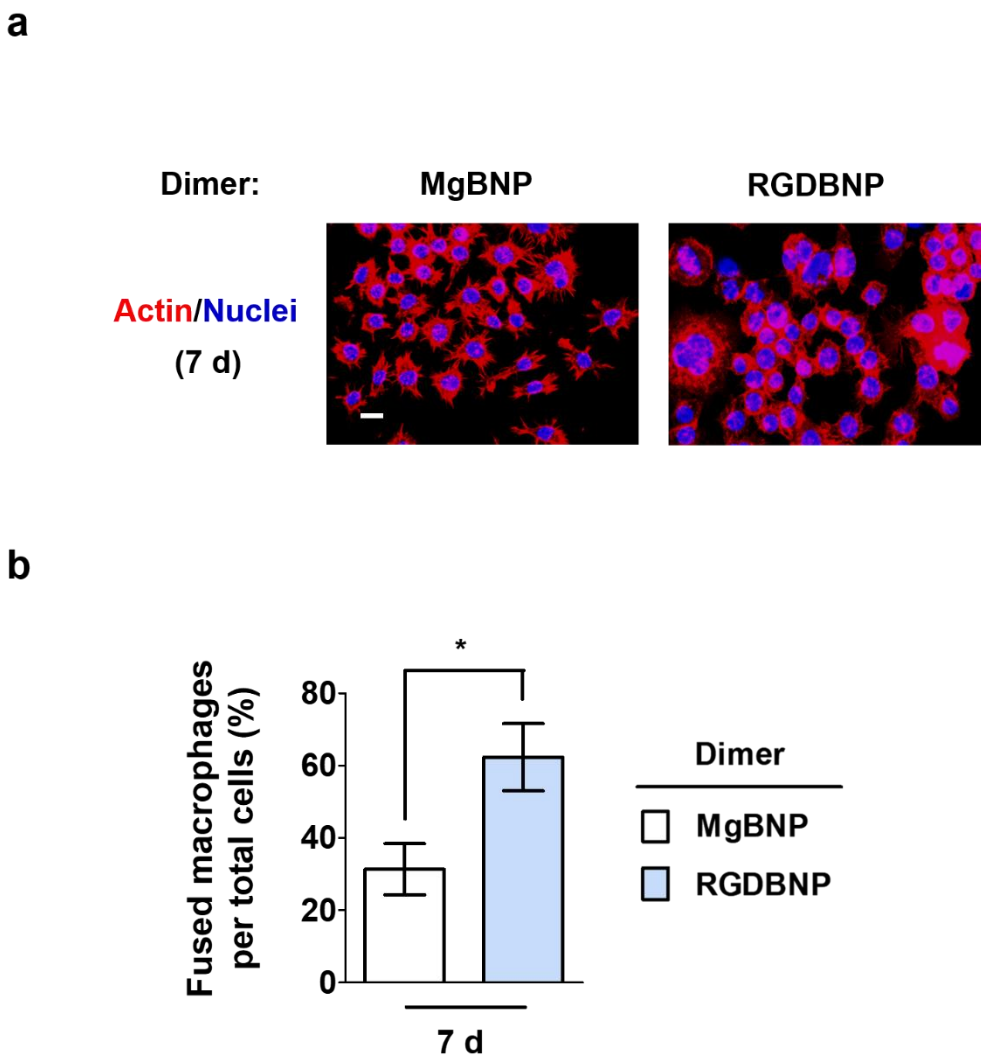

Supplementary Fig. 23 The dimeric nanoassembly presenting RGD-BP stimulates the fusion of macrophages. **a** Fluorescently images of macrophages stained for actin (in red) and nuclei (in blue), which adhered to the MgBNP or RGDBNP dimeric substrate after 7 d under culture in the M2-type-polarizing medium, and **b** corresponding quantification of fused macrophages. Scale bar is 50 µm. Data are means ± s.d. (n=3). *P < 0.05 (two-tailed Student's t-test).

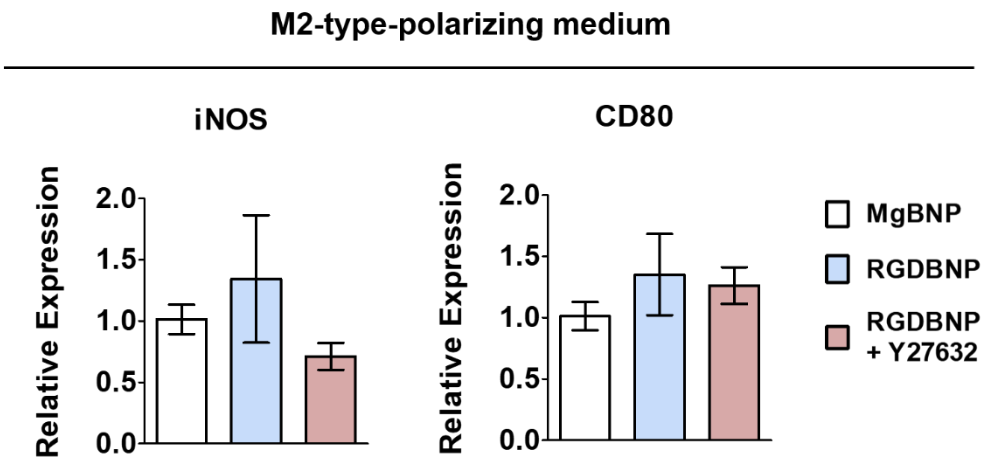

Supplementary Fig. 24 Expression levels of M1-type markers (iNOS and CD80 gene) determined by RT-qPCR, for adherent macrophages after culture in M2-type-polarizing medium with or without ROCK inhibition. Cells were cultured for 36 h (the first 12 h under culture in basal medium, followed by 24 h under culture in M2-type-polarizing medium). The dimeric nanoassembly of Mg^{2+}-BP NPs (MgBNP dimer) or RGD-Mg^{2+}-BP NPs (RGDBNP dimer) was directed after 0 h under culture. The "RGDBNP" dimer was also cultured in the presence of the ROCK inhibitor, Y27632 (RGDBNP + Y27632). Data are means ± s.e.m. (n=3). Gene expression is presented as the relative fold expression of the target genes (iNOS or CD80) after normalization to those in the MgBNP dimer.

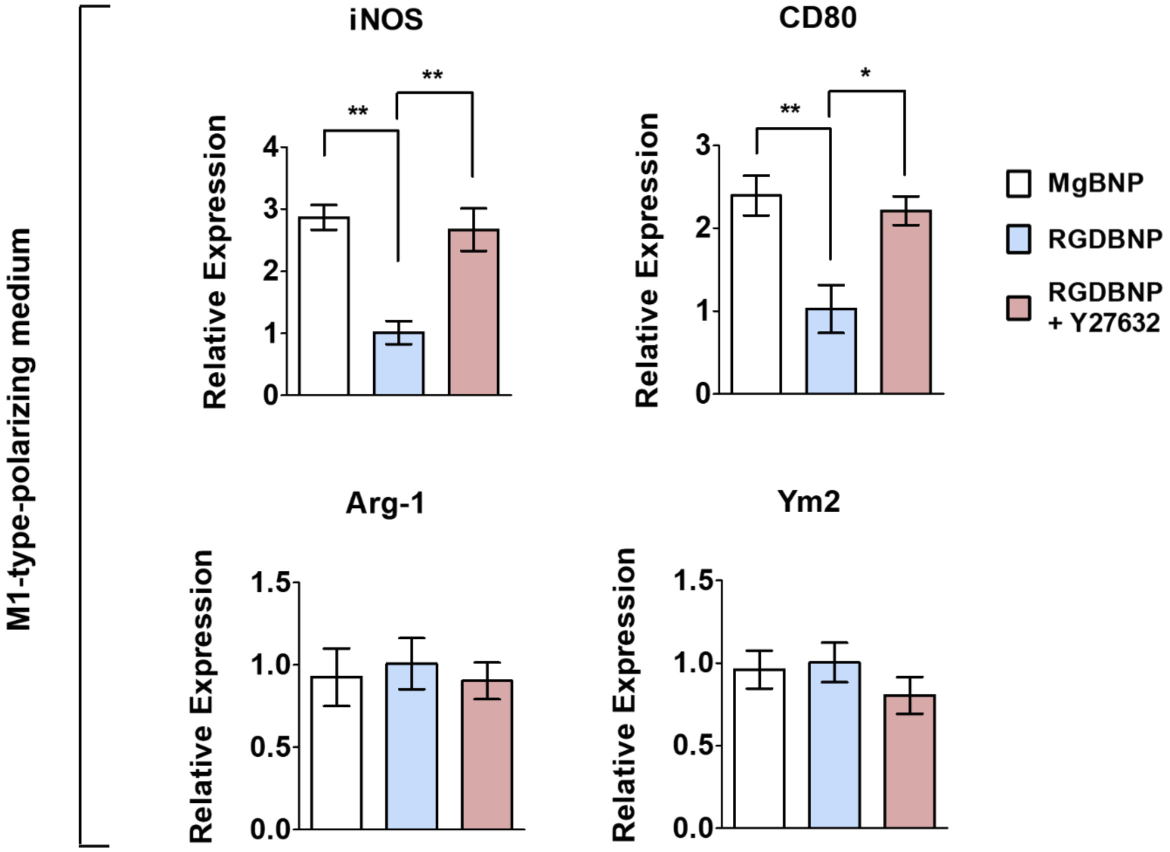

Supplementary Fig. 25 Expression levels of M1-type markers (iNOS and CD80 gene) and M2-type markers (Arg-1 and Ym2 genes) determined by RT-qPCR, for adherent macrophages after culture in M1-type-polarizing medium with or without ROCK inhibition. Cells were cultured for 36 h (the first 12 h under culture in basal medium, followed by 24 h under culture in M1-type-polarizing medium. The dimeric nanoassembly of Mg^{2+}-BP NPs (MgBNP dimer) or RGD-Mg^{2+}-BP NPs (RGDBNP dimer) was directed after 0 h under culture. The RGDBNP dimer was also cultured with the ROCK inhibitor, Y27632 (RGDBNP + Y27632). Gene expression is presented as the relative fold expression of the target genes (iNOS, CD80, Arg-1, or Ym2) after normalization to those in the RGDBNP dimer. Data are means ± s.e.m. (n=3). $^{*}P < 0.05$, $^{**}P < 0.01$ (ANOVA).

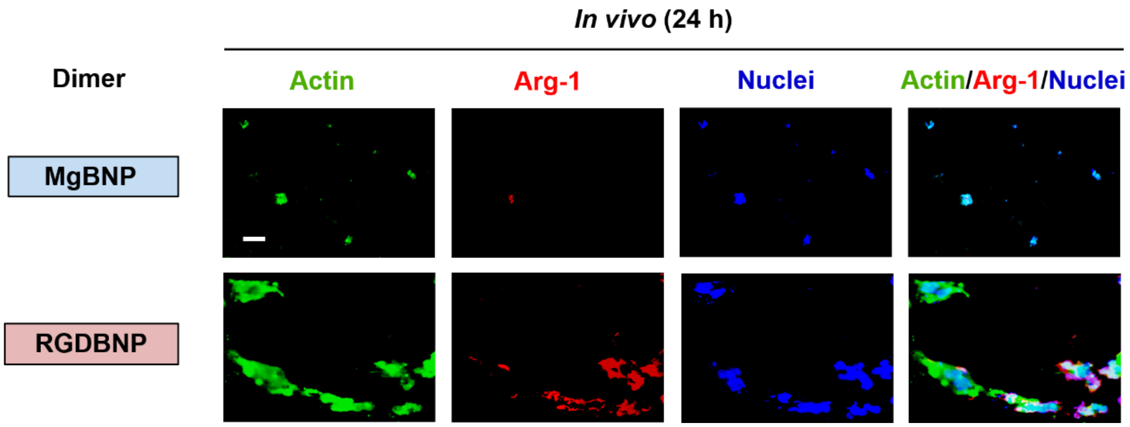

Supplementary Fig. 26 M2-type polarization of *in vivo* adherent host macrophages under dimeric nanoassembly. Fluorescent images of *in vivo* adherent host cells immunostained for actin (in green), Arg-1 (in red), and nuclei (in blue) at 24 h post-implantation of the BP-AuNP-grafted substrate, which was subjected to dimeric nanoassembly by the administration of BP, Mg^{2+}, or RGD-BP immediately following implantation. Scale bar is 20 μm.

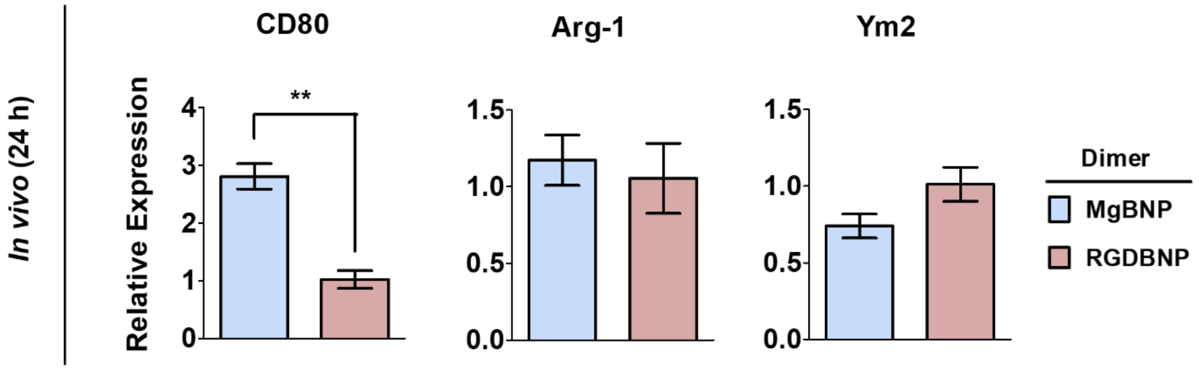

Supplementary Fig. 27 The dimeric nanoassembly bearing RGD-BP inhibits M1-type polarization of *in vivo* adherent host cells. Expression of the M1-type marker (CD80 gene) and M2-type markers (Arg-1 and Ym2 genes) determined by RT-qPCR, for adherent host cells at 24 h post-implantation of the BP-AuNP-grafted substrate, which was subjected to dimeric nanoassembly by the administration of BP, Mg^{2+}, or RGD-BP immediately following implantation. Gene expression data are shown as the relative fold expression of the gene of interest (CD80, Arg-1, and Ym2) after normalization to their expression in the RGDBNP dimer. Data are means ± s.e.m. (n=3). **$P < 0.01$ (two-tailed Student's t-test).

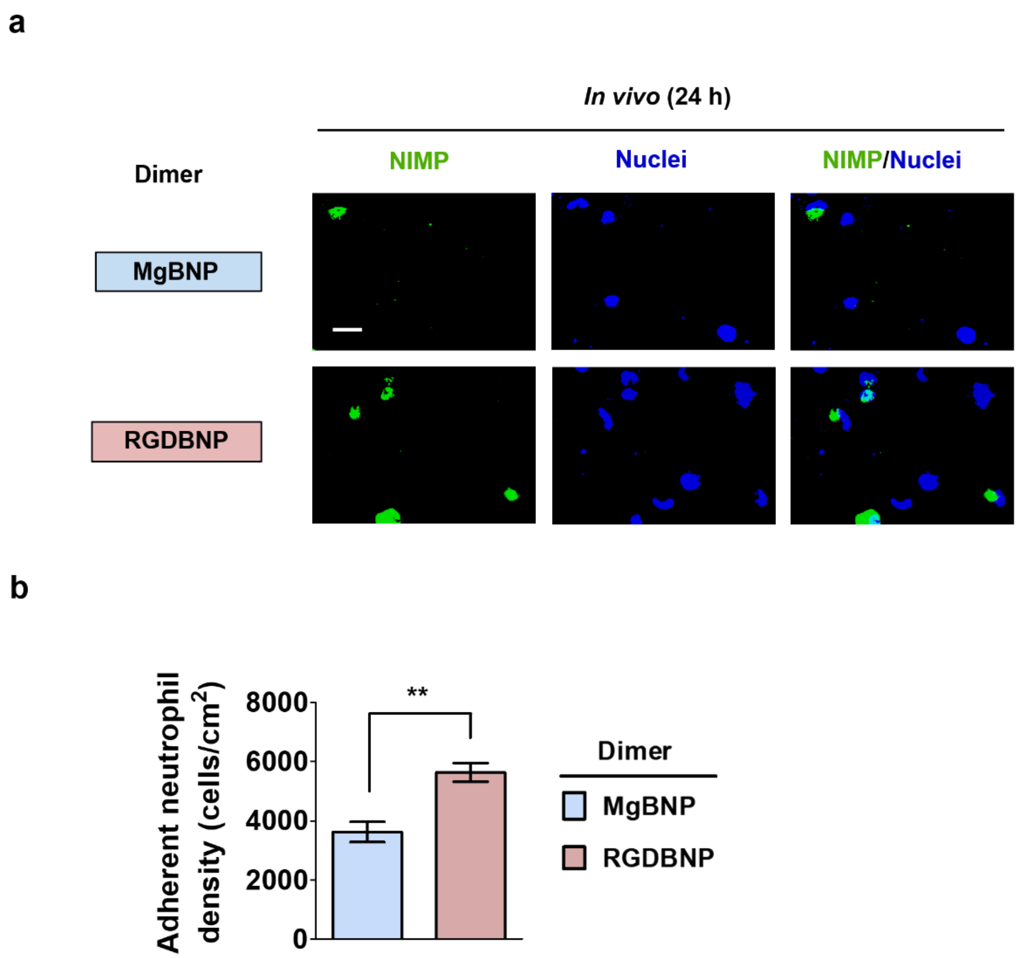

Supplementary Fig. 28 The adhesion of host neutrophils *in vivo* under dimeric nanoassembly. **a** Fluorescent images of adherent host cells immunostained for NIMP-R14 (in green) and nuclei (in blue) at 24 h post-implantation of the BP-AuNP-grafted substrate, which was subjected to dimeric nanoassembly by the administration of BP, Mg^{2+}, or RGD-BP immediately following implantation. Scale bar is 20 µm. **b** Corresponding quantification of the densities of *in vivo* adherent host neutrophils. Data are means ± s.e.m. (n=4). **P < 0.01 (two-tailed Student's t-test).

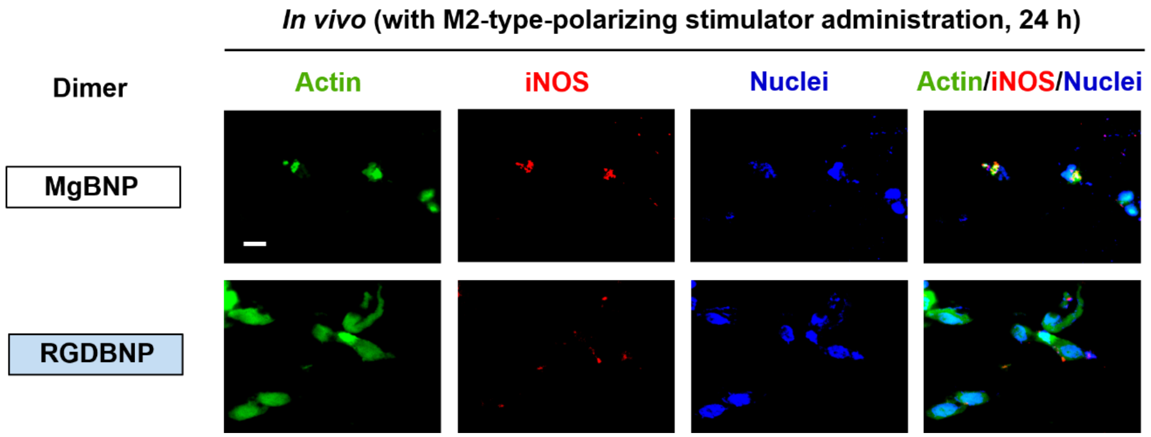

Supplementary Fig. 29 The dimeric nanoassembly presenting RGD-BP with M2-type-polarizing stimulators suppresses M1-type polarization of *in vivo* adherent host macrophages. Fluorescent images of *in vivo* adherent host cells immunostained for actin (in green), iNOS (in red), and nuclei (in blue) at 24 h post-implantation of the BP-AuNP-grafted substrate, which was subjected to dimeric nanoassembly by the administration of BP, Mg^{2+}, or RGD-BP and then M2-type-polarizing stimulators (IL-4 and IL-13 cytokines) immediately following implantation. Scale bar is 20 μm.

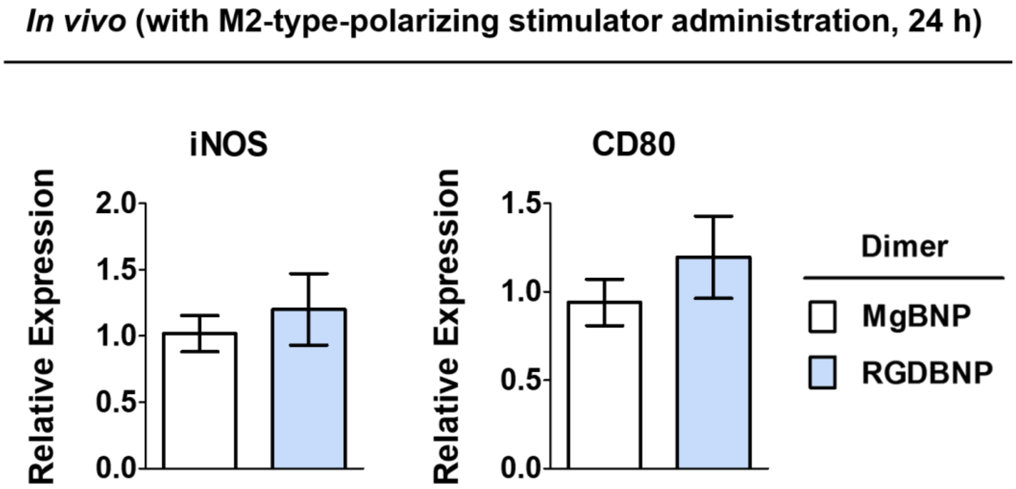

Supplementary Fig. 30 Quantitative analysis of the M1-type polarization of adherent host cells *in vivo* under dimeric nanoassembly with M2-type-polarizing stimulators. Expression of M1-type markers (iNOS and CD80 genes) determined by RT-qPCR, for adherent host cells at 24 h following the implantation of the BP-AuNP-grafted substrate, which was subjected to dimeric nanoassembly by the administration of BP, Mg^{2+}, or RGD-BP and then M2-type-polarizing stimulators (IL-4 and IL-13 cytokines) immediately following implantation. Data are means ± s.e.m. (n=3). Gene expression is presented as the relative fold expression of the target genes (iNOS and CD80) after normalization to their expression in the MgBNP dimer.

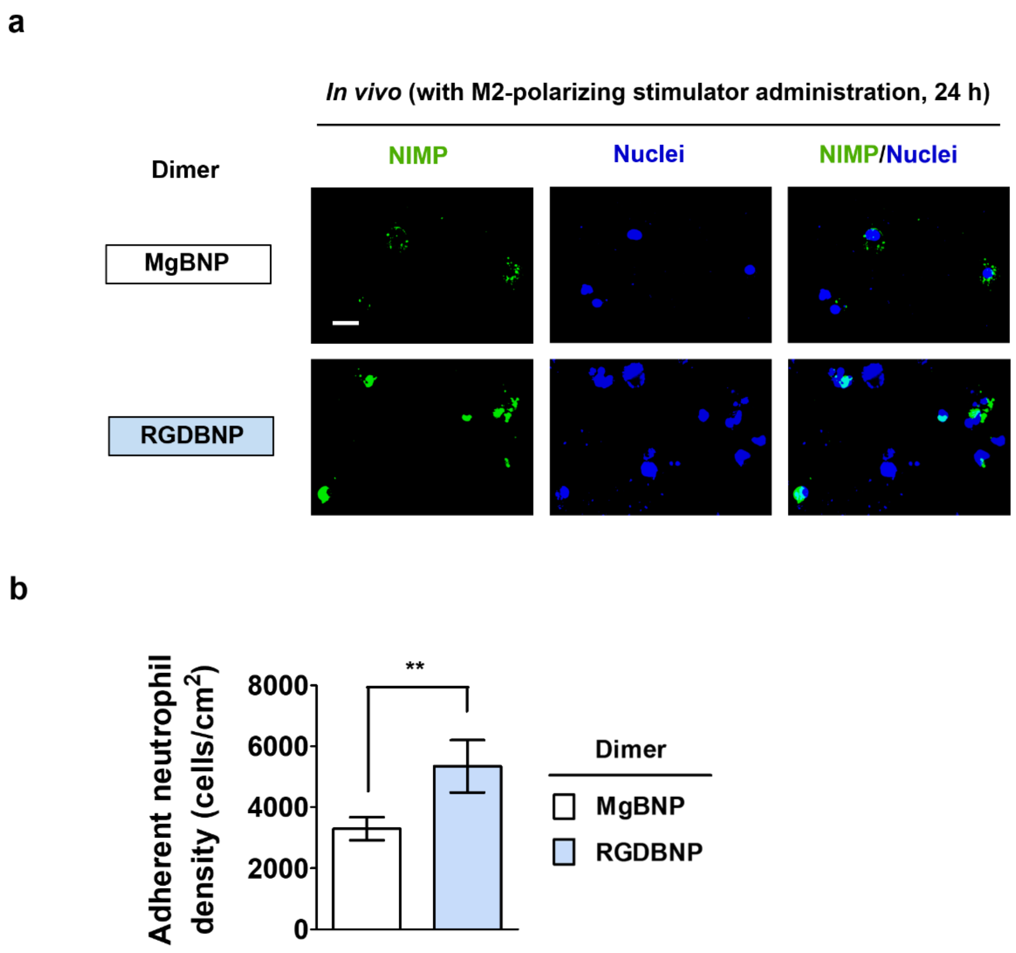

Supplementary Fig. 31 The adhesion of host neutrophils *in vivo* under dimeric nanoassembly in the presence of M2-type-polarizing stimulators. **a** Fluorescent images of adherent host cells immunostained for NIMP-R14 (in green) and nuclei (in blue) at 24 h post-implantation of the BP-AuNP-grafted substrate, which was subjected to dimeric nanoassembly by the administration of BP, Mg^{2+}, or RGD-BP and then M2-type-polarizing stimulators (IL-4 and IL-13 cytokines) immediately following implantation. Scale bar is 20 µm. **b** Corresponding quantification of the densities of *in vivo* adherent host neutrophils. Data are means ± s.d. (n=4). **P < 0.01 (two-tailed Student's t-test).

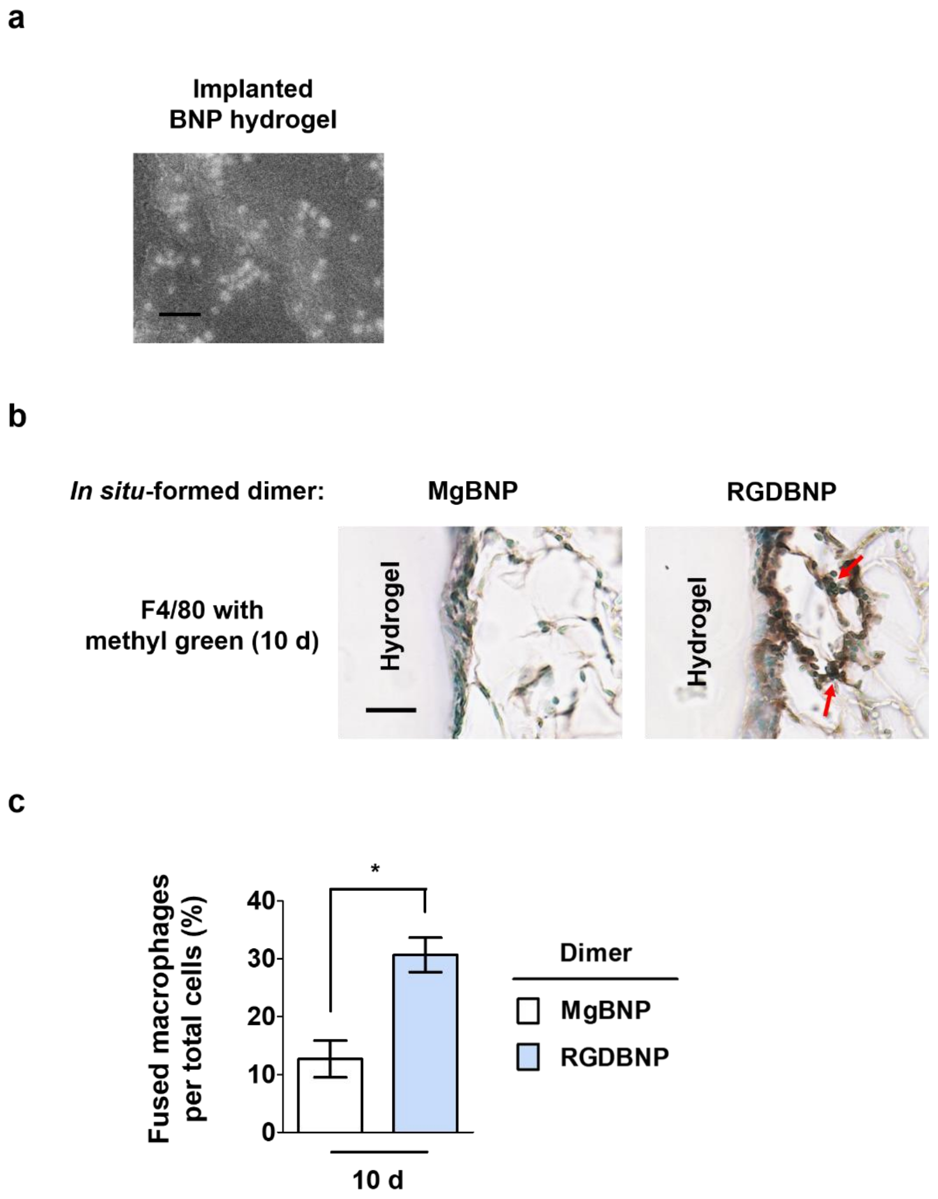

Supplementary Fig. 32 The formation of RGD-presenting dimers promotes macrophage fusion *in vivo*. **a** Scanning electron microscopic image of the implanted BNP hydrogel substrate, which was then subjected to the *in situ* formation of the MgBNP or RGDBNP substrates by the administration of BP, Mg^{2+}, or RGD-BP followed by M2-type-polarizing stimulators (IL-4 and IL-13 cytokines). Scale bar is 50 nm. Five mice were used per group. **b** Immunohistochemically stained images for F4/80 with methyl green counterstaining and **c** corresponding quantification of fused macrophages at 10 d post-implantation. Scale bar is 50 µm. Red arrows indicate fused macrophages. Data are means ± s.e.m. (n=3). *P < 0.05 (two-tailed Student's t-test).
