# Supplementary material for: Immunoregulation of macrophages by dynamic ligand presentation via ligand–cation coordination
Source: Nat Commun. 2019 Apr 12;10:1696. doi: 10.1038/s41467-019-09733-6 (PMC6461616; doi:10.1038/s41467-019-09733-6)
Supplement: Supplementary file 2 — Description of Additional Supplementary Files [file 41467_2019_9733_MOESM2_ESM.pdf]

Description of Additional Supplementary Files

File Name: Supplementary Movie 1
Description: The real-time fluorescent monitoring of macrophage detachment. The Mg^{2+}-BP nanoparticles in the dimers dissolve and disassemble under gentle mixing of EDTA, which mediate macrophage detachment. The movie plays for 10 s, which corresponds to approximately 10 min in real-time.
